# Glucocorticoid-induced phosphorylation by CDK9 modulates the coactivator functions of transcriptional cofactor GRIP1 in macrophages

**DOI:** 10.1038/s41467-017-01569-2

**Published:** 2017-11-23

**Authors:** David A. Rollins, Joubert B. Kharlyngdoh, Maddalena Coppo, Bowranigan Tharmalingam, Sanda Mimouna, Ziyi Guo, Maria A. Sacta, Miles A. Pufall, Robert P. Fisher, Xiaoyu Hu, Yurii Chinenov, Inez Rogatsky

**Affiliations:** 1 Graduate Program in Immunology and Microbial Pathogenesis, Weill Cornell Graduate School of Medical Sciences, 1300 York Avenue, New York, NY 10021 USA; 2 Hospital for Special Surgery Research Institute, The David Rosensweig Genomics Center, 535 East 70th Street, New York, NY 10021 USA; 3 Institute for Immunology and School of Medicine, Tsinghua University, Beijing, 100084 China; 4 Weill Cornell/ Sloan Kettering/ Rockefeller Tri-Institutional MD-PhD Program, 1300 York Avenue, New York, NY 10021 USA; 5 Department of Biochemistry, University of Iowa, 51 Newton Road, Iowa City, IA 52242 USA; 6 Department of Oncological Sciences, Icahn School of Medicine at Mount Sinai, One Gustave L. Levy Place, New York, NY 10029 USA

## Abstract

The glucocorticoid (GC) receptor (GR) suppresses inflammation by activating anti-inflammatory and repressing pro-inflammatory genes. GR-interacting protein-1 (GRIP1) is a GR corepressor in macrophages, however, whether GRIP1 mediates GR-activated transcription, and what dictates its coactivator versus corepressor properties is unknown. Here we report that GRIP1 loss in macrophages attenuates glucocorticoid induction of several anti-inflammatory targets, and that GC treatment of quiescent macrophages globally directs GRIP1 toward GR binding sites dominated by palindromic GC response elements (GRE), suggesting a non-redundant GRIP1 function as a GR coactivator. Interestingly, GRIP1 is phosphorylated at an N-terminal serine cluster by cyclin-dependent kinase-9 (CDK9), which is recruited into GC-induced GR:GRIP1:CDK9 hetero-complexes, producing distinct GRE-specific GRIP1 phospho-isoforms. Phosphorylation potentiates GRIP1 coactivator but, remarkably, not its corepressor properties. Consistently, phospho-GRIP1 and CDK9 are not detected at GR transrepression sites near pro-inflammatory genes. Thus, GR restricts actions of its own coregulator via CDK9-mediated phosphorylation to a subset of anti-inflammatory genes.

## Abstract (web summary)

Glucocorticoid reduces inflammation by both inducing anti-inflammatory genes and suppressing pro-inflammatory genes, but how these two functions are dictated is unclear. Here the authors show that phosphorylated glucocorticoid receptor-interacting protein 1 (GRIP1) serves as a coactivator for this response in macrophage.

## Introduction

Inflammation is a host response to infection or injury triggered when innate immune cells such as macrophages identify pathogenic molecules via pattern recognition receptors (for example Toll-like receptors (TLR)) and initiate an inflammatory gene expression program executed primarily by the effector transcription factors NF-κB and AP1^1^. Although normally protective, inflammation is potentially dangerous to the host and contributes to the pathogenesis of numerous human pathologies including rheumatoid arthritis, lupus, multiple sclerosis, and others^2^. Hence, several mechanisms have evolved that limit inflammatory responses at the local and systemic level. In particular, inflammatory mediators trigger the production of glucocorticoids (GC), steroid hormones with profound anti-inflammatory properties^3^. GCs signal through the GC receptor (GR), a ligand-dependent transcription factor of the nuclear receptor (NR) superfamily that, upon hormone binding, is recruited to genomic GC response elements (GRE) to regulate gene expression^4^. Several classes of GREs have been described: at palindromic GREs, GR binds DNA directly and typically activates target genes, including anti-inflammatory mediators such as *Dusp1* and *Gilz* ^5,6^. Equally common are “tethering” GREs, where GR associates with other DNA-bound regulators, most prominently NF-κB or AP1, and represses their pro-inflammatory target genes (including *Tnf* and *Il1b*)^7^. Activation of anti-inflammatory and repression of pro-inflammatory genes both critically contribute to the therapeutic efficacy of GCs, which have been exploited in medicine for over half a century^8–11^.

To exert transcriptional effects, GR assembles multi-subunit protein-DNA complexes composed of numerous coregulators. In addition to linking sequence-specific transcription factors with basal machinery and chromatin, coregulators integrate diverse environmental signals and transcription programs to ultimately elicit an appropriate physiological response. Prominent NR cofactors are the members of NR coactivator (NCoA)1-3 p160 family, which provide binding platforms for secondary coregulators and chromatin modifiers, for example, histone acetyltransferases (CBP/p300) and methyltransferases (G9a, CARM1) (reviewed in refs. ^12,13^). NCoA1-3 were originally identified as interactors of agonist-bound NRs and found to serve as coactivators in overexpression studies^14–17^. Functional redundancy between p160s has been supported by ~60% similarity across their NR-interaction domain as well as their activation domains 1 and 2 that bind CBP/p300 and CARM1, respectively^13,18,19^. Yet, subsequent in vivo studies with mice revealed non-redundant, sometimes antagonistic functions for these proteins in immune, metabolic and reproductive physiology and pathology (reviewed in refs. ^12,13,19^). Given the ability of these coregulators to cooperate with numerous NRs and non-receptor transcription factors, however, the exact transcriptional pathways responsible for the observed unique phenotypes of p160 deficiencies in vivo in different cell types remain largely undefined.

In regards to GC signaling, NCoA2 (also known as GR-interacting protein (GRIP)1)^15,16^ has emerged as a unique family member that, unlike other p160s, associates with GR at “tethering” sites and facilitates GC repression of AP1- and NF-κB-driven targets^20,21^. Indeed, GRIP1 deletion in macrophages derepresses genes encoding inflammatory mediators in vitro and sensitizes mice to TLR4-induced systemic inflammation in vivo^22^. In contrast, whether GRIP1 contributes to GR-mediated gene activation in macrophages, or in pertinent physiological settings in a manner that cannot be compensated by other p160s has never been evaluated. Given that intracellular levels of individual p160 family members vary among different cell types, and that the abundance of GRIP1 in murine macrophages is low^23^, it is possible that GRIP1 recruitment to distinct GR transcription complexes is stochastic and transcriptional complexes effectively compete with each other for limiting amounts of GRIP1. Alternatively, there could be signals that direct GRIP1 to specific genomic GR binding sites (GBS) or gene classes. Finally, it remains unexplored how the stimulatory vs. inhibitory properties of this bifunctional coreguator are specified. In principle, the allosteric effects of interacting complex components may dictate GRIP1 function as a coactivator or a corepressor; it is also possible that additional regulatory inputs constrain GRIP1 activities to operating as one or the other.

Our group previously reported that, in response to GCs, GRIP1 is phosphorylated at four serines, S469, S487, S493, and S499, outside any characterized functional domain, in a GR:GRIP1 interaction-dependent manner^22,24^. Post-translational modifications (PTM) of proteins are known to affect their biochemical and functional characteristics including stability, subcellular localization and protein:protein interactions. In the case of transcriptional cofactors, such as the p160s, PTMs would be expected to potentiate or attenuate gene expression programs (reviewed in refs. ^12,13,19^),conceivably, in a cell context-specific manner. Nonetheless, the importance of GRIP1 phosphorylation in inflammation and biology of macrophages, a cell type in which GR actions bear therapeutic value, has not been reported.

Here we investigate the function of GRIP1 as a GR coactivator in mouse and human macrophages by assessing GR-driven gene expression after GRIP1 deletion and global GR and GRIP1 genome-wide distribution. We further present evidence that GR, by recruiting the GRIP1 kinase, sets up a versatile and precise mechanism to ensure GRIP1 modification in a GBS-specific manner. Finally, we dissect the functional implications of GC-induced GRIP1 phosphorylation with respect to its coactivator versus corepressor properties in GR-regulated gene expression in macrophages.

## Results

### GRIP1 mediates GC induction of anti-inflammatory genes

**Fig. 1:**
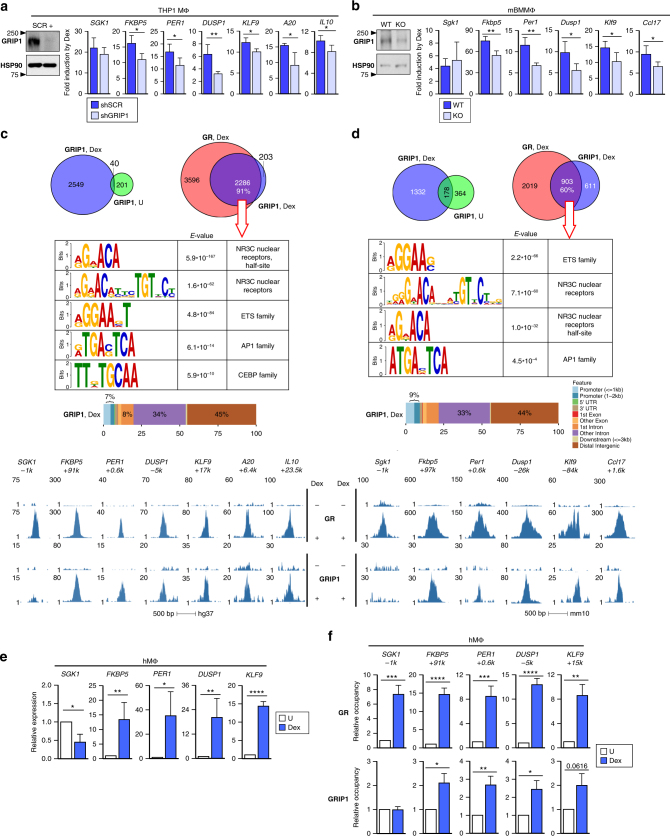
Macrophage GRIP1 is a GR coactivator of anti-inflammatory genes. GRIP1 was depleted in **a** THP1 cells (using a human-specific small-hairpin (sh)GRIP1 vs. scrambled (shSCR) control) or **b** mouse BMMΦ (from GRIP1 KO vs. WT control mice), and the induction of indicated genes by dexamethasone (Dex; 100 nM, 2 h) was analyzed by RT-qPCR, normalized to the expression of β-actin and expressed relative to untreated (=1). GRIP1 depletion efficiency is shown by immunoblotting with HSP90 as a loading control on the left (quantified in Supplementary Fig. 1b; see Supplementary Fig. 10a for full-size bots). ChIP-seq for GR and GRIP1 was performed in THP1 cells (**c**) or mBMMΦ (**d**) treated ±Dex for 1 h (or 45 min for GR in BMMΦ), as indicated, and peaks were called as described in Methods. (Top) The overlap between peaks for GRIP1±Dex or GRIP1 and GR (Venn diagrams) was determined using *subsetByOverlap* function from GenomicRanges package (Bioconductor) with the minimum overlap of 1 bp (see “Methods”). (Middle) Ab initio sequence motif overrepresentation in GR–GRIP1 overlapping peaks was determined using MEME-ChIP. Genomic location of GRIP1 binding sites relative to known genomic features was determined by *ChIPpeakAnno* (Bioconductor). The union of peaks for two GRIP1 replicas in each cell type, the consensus for two THP1 GR replicas and a single mBMMΦ GR experiment are shown (Supplementary Figs. 2–4). (Bottom) Read density profiles of genes analyzed in **a**, **b** show peaks for GR and GRIP1. **e** CD14^+^hMΦ were untreated (U) or treated with Dex for 2 h, and the expression of representative genes was measured as in **a**. **f** GR and GRIP1 occupancy at relevant sites in hMΦ was assessed by ChIP-qPCR (normalized to the 28s rDNA housekeeping gene, untreated=1). Samples in **a**, **b** and **e**, **f** were compared by unpaired two-tailed Student’s *t*-test. Shown are means+standard deviation (SD) error bars (*n* ≥ 3, *P < 0.05, **P < 0.01, *** *P* < 0.001, **** *P* < 0.0001)

The ability of GR to induce genes encoding anti-inflammatory mediators in macrophages critically contributes to the efficacy of GC therapies^8–11^. Indeed, in human THP1 differentiated macrophage-like cells (THP1 MΦ) and in mouse primary bone marrow-derived (BM)MΦ, a 2-h treatment with the synthetic GC dexamethasone induced expression of the canonical GR target Fkbp5 as well as genes with known anti-inflammatory function: *SGK1*, *PER1, DUSP1, KLF9*, *A20*, *GILZ*, *IL10* and *Sgk1*, *Per1*, *Dusp1, Klf9, Ccl17* and *Gilz* in human and mouse, respectively^7,25–30^ (Supplementary Fig. 1a). To test whether the p160 coregulator GRIP1 is necessary for this induction, THP1 MΦ stably depleted of GRIP1 via lentiviral-delivered small-hairpin (sh)RNA KD (shGRIP1; Supplementary Fig. 1b, left) or BMMΦ from LysM-Cre*GRIP1* ^*fl/fl*^ mice (GRIP1 KO^31^) and their respective scrambled-transduced (shSCR) or GRIP1^fl/fl^ (WT) controls (Fig. 1a, b, left; see Supplementary Fig. 1b for the quantification of GRIP1 protein and RNA) were treated with dexamethasone for 2 h and gene expression compared. Figure 1a, b (right) show that GRIP1 depletion attenuated GC induction of all GR targets examined except for *SGK1* (and anti-inflammatory gene *MT2A* ^32^, Supplementary Fig. 1c) in THP1 MΦ and *Sgk1* in BMMΦ, whose regulation by dexamethasone appeared GRIP1-independent. Importantly, differences in GC induction of genes were not due to altered basal expression (Supplementary Fig. 1d). Together, these results suggested that endogenous GRIP1 performs non-redundant functions in GR-activated gene expression in macrophages.

Because no genome-wide analysis of GR and GRIP1 co-distribution in macrophages after acute GC exposure has been reported, we performed GR and GRIP1 ChIP-seq in macrophages treated with Dex for 45–60 min and compared their localization. Correlation between reads found in GR and GRIP1 peaks in individual replicates indicated considerable reproducibility of ChIP-seq experiments (Supplementary Figs. 2a, 3b and 4b). Consistent with ligand activation, very few GR binding events were detected in untreated cells (5 and 77 in THP1 MΦ and 103 in BMMΦ), whereas dexamethasone treatment triggered rapid GR mobilization with several thousand GBS detectable (Fig. 1c, d, Supplementary Figs. 2a and e, 3a and 4a). Even though GRIP1 does not bind GCs, dexamethasone treatment resulted in a dramatic increase in GRIP1 binding sites (~tenfold for THP1 MΦ and ~threefold for BMMΦ; Fig. 1c, d, top; Supplementary Figs. 3a, b, 4a, b). Strikingly, there was minimal overlap between the GRIP1 peaks ± dexamethasone (40 peaks in THP1 MΦ and 178 peaks in BMMΦ, Supplementary Figs. 3b, 4b). Moreover, under liganded conditions, 2286 (91%) and 903 (60%) of GRIP1 peaks in THP1 MΦ and BMMΦ, respectively, overlapped with GR peaks (Fig. 1c, d, top). These overlapping peaks in THP1 MΦ exhibited higher pileup values (Supplementary Fig. 3d), suggesting that these sites either have a higher affinity for GR or are in a more permissive chromatin environment.

Ab initio motif discovery/overrepresentation analyses with MEME-ChIP at GRIP1 sites overlapping GBS or of the GBS themselves in dexamethasone -treated macrophages showed an enrichment of NR3C sites, which includes GREs as principal binding motifs, within the peak (Fig. 1c, d, middle; Supplementary Fig. 2b, f) and near peak summits (Supplementary Figs. 2c, g and 3g). ETS, AP1, and CEBP motifs were also enriched within GBS with the ETS motif enriched near the summit in BMMΦ (Supplementary Fig. 2g). Of note, GBS motif analysis in THP1 MΦ identified a putative composite site containing a GRE half-site and IRF-like binding motif (Supplementary Fig. 2b), suggesting potential interactions between these classes of transcription factors. Further supporting a model whereby ligand dedicates GRIP1 to GR signaling, GRIP1 binding distribution mirrored that of GR—primarily distal intergenic (42–46%) and intronic (32–34%) (Fig. 1c, d, Supplementary Fig. 2a, e)—similar to GR binding distribution in other cell types^33^. Interestingly, pathway analysis of genes associated with GR–GRIP1 overlapping peaks revealed immune-related signatures in both human and mouse cells, in addition to cell adhesion pathways seen in THP1 MΦ and hypoxia and circadian clock signatures in BMMΦ (Supplementary Figs. 3h, 4f). Consistently, pathway analysis of GR peaks further supported extensive involvement of GR in regulating inflammation and innate immunity (Supplementary Fig. 2d, h).

In sharp contrast, there was a minimal overlap of GRIP1 peaks in untreated MΦ with those of liganded GR: 32 (13.4%) in THP1 MΦ and 13 (0.02%) in BMMΦ (Supplementary Figs. 3b, 4b). Notably, motif enrichment analysis for GRIP1 binding peaks in untreated MΦ revealed the overabundance of low-complexity sequences (centromeric, tri- and tetra-nucleotide repeats; Supplementary Figs. 3f, 4d) that likely represent non-specific or low affinity binding or artifacts of peak calling^34^. Further, we analyzed GRIP1-unique peaks in dexamethasone-treated THP1 MΦ (203 peaks in Fig. 1c and Supplementary Fig. 3c) and BMMΦ (611 peaks in Fig. 1d and Supplementary Fig. 4c). In both cell types, these peaks were enriched for low-complexity sequences with similarity to Tcf7, Tbp, and ETS binding sites (Supplementary Figs. 3h, 4e). GRIP1-unique peaks were also smaller (Supplementary Fig. 3d), suggesting either weaker DNA binding affinity for the factor recruiting GRIP1 or non-permissive chromatin. This result, combined with the lack of sizable overlap of these peaks between GRIP1 replicas (Supplementary Figs. 3c, 4c, bottom), is consistent with GRIP1 binding being largely signal (in our case, liganded GR)-driven in both species.

Given our results demonstrating the function of GRIP1 in GR target gene activation, we evaluated ChIP-seq read distributions at GR targets analyzed in Fig. 1a, b. Dexamethasone -induced GR and GRIP1 peaks colocalized at all GBS, except for the GBS at −1 kb of *SGK1* or *Sgk1* (Fig. 1c, d, bottom), whose induction was GRIP1-independent. GR and GRIP1 ChIP-seq results from both cell types were validated by ChIP-qPCR (Supplementary Fig. 1e). Together, these results implicate GRIP1 as a key GR coactivator, whose loss is responsible for the impaired GC induction of genes in GRIP1-depleted macrophages (Fig. 1a, b).

Although the THP1 cell line is commonly used to study MΦ functions^35^, we corroborated our findings in primary human macrophages differentiated in vitro from CD14+ monocytes. Figure 1e shows that a 2-h dexamethasone treatment of hMΦ induces *FKBP5, PER1, DUSP1*, and *KLF9*, and negatively regulates *SGK1*. Furthermore, GR and GRIP1 colocalized at the *FKBP5, PER1, DUSP1*, and *KLF9* GBS, whereas only GR occupied the *SGK1* GBS (Fig. 1f), recapitulating the THP1 results.

### CDK9 phosphorylates GRIP1 in a GC-dependent manner

**Fig. 2:**
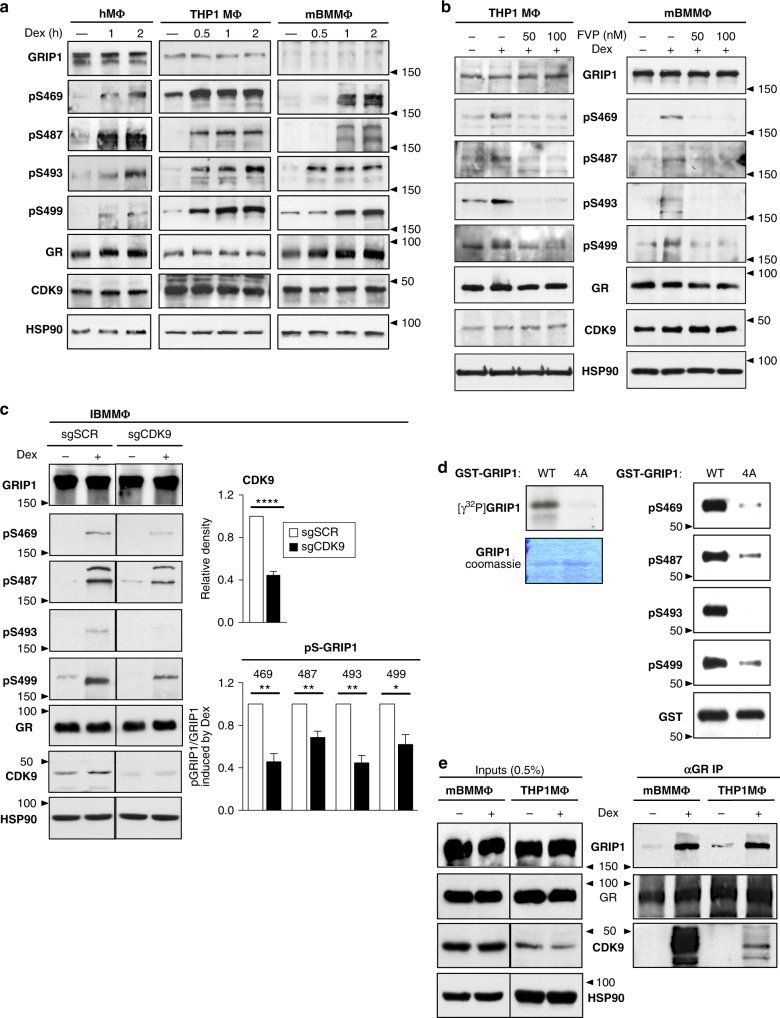
GRIP1 is a substrate for dexamethasone (Dex)-induced phosphorylation by CDK9 in macrophages. **a** hMΦ, THP1 cells or BMMΦ were treated with Dex for the times indicated and the levels of GRIP1 (total or phosphorylated (p) at S469, S487, S493, and S499), GR, CDK9 and HSP90 as a loading control were assessed by immunoblotting (quantified in Supplementary Fig. 5a). **b** THP1 cells or mBMMΦ were treated with Dex±flavopiridol (FVP) at indicated concentrations for 2 h and GRIP1 phosphorylation was visualized by immunoblotting (quantified in Supplementary Fig. 5b). **c** Immortalized BMMΦ were depleted of CDK9 (single guide (sg)CDK9 vs. scrambled sgSCR control) as described in Methods and treated ±Dex for 1 h. The levels of GRIP1, phospho-(p)GRIP1, GR, CDK9 and HSP90 were assayed by western blot and quantified by densitometry; CDK9 levels or ratios of pGRIP1/GRIP1 in sgCDK9 cells are expressed relative to those in sgSCR cells (=1). Samples were compared by unpaired, two-tailed Student’s *t*-test; means + standard error of the mean (SEM) are shown (*n* ≥ 3, *P < 0.05, **P < 0.01). **d** *E*. *coli*-produced affinity-purified GST–GRIP1_322-631_ WT and S469A/S487A/S493A/S499A (4A) were subjected to in vitro kinase assays with baculovirus-expressed cyclin T1-CDK9. GRIP1 phosphorylation was assessed by autoradiography (left) and immunoblotting (right) with Coomassie blue staining (left) or immunoblotting for GST-tag (right) showing equal loading. **e** BMMΦ or THP1 cells were treated ±Dex for 1 h, double cross-linked and GR immunoprecipitations (IP) performed (see “Methods”). Inputs and IPs were immunoblotted for GRIP1, GR and CDK9 (with HSP90 as an input loading control). Full-size western blots are shown in Supplementary Fig. 10a–e

We previously showed that GRIP1 is phosphorylated at a cluster of serines—S469, S487, S493, and S499—in a dexamethasone- and GR:GRIP1 interaction-dependent manner^24^. Because that work was performed primarily in a U2OS osteosarcoma cell line overexpressing GR^36^, we questioned whether phosphorylation was conserved in macrophages, a key innate immune cell type in which anti-inflammatory actions of GCs are physiologically relevant. Immunoblotting of hMΦ, THP1 MΦ and BMMΦ using phosphosite-specific antibodies to pS469, pS487, pS493, and pS499^24^ revealed dexamethasone-induced phosphorylation of each serine without a change in total GRIP1 or GR levels (Fig. 2a; quantified in Supplementary Fig. 5a). Interestingly, these serines were phosphorylated with varied kinetics depending on specific site and species.

To identify the kinases potentially responsible for GRIP1 phosphorylation in macrophages, we tested a panel of kinase inhibitors and found that flavopiridol (FVP), a CDK9 inhibitor, abrogated dexamethasone-dependent GRIP1 phosphorylation in both THP1 MΦ and BMMΦ (Fig. 2b, quantified in Supplementary Fig. 5b). Because chemical inhibition may suffer from limited selectivity, we knocked down CDK9 in immortalized BMMΦ (IBMMΦ) via lentiviral transduction of CRISPR–Cas9 gRNA against CDK9 (Supplementary Fig. 5c). Notably, CDK9 is required for transcriptional elongation by RNA polymerase II (Pol II)^37,38^, and its complete ablation would adversely affect cell viability. Strikingly, we found that CDK9 KD efficiency of ~60% correlated well with the relative loss of dexamethasone-induced GRIP1 phosphorylation (Fig. 2c). To determine whether CDK9 can directly utilize GRIP1 as a substrate, we performed in vitro kinase assays using baculovirus-expressed cyclin T1/CDK9 complex and a recombinant GST–GRIP1_322–631_ fragment containing the four target serines or mutant alanines (4A). CDK9 phosphorylated WT GRIP1_322–631_, but not a 4A mutant (Fig. 2d), establishing that CDK9 can directly phosphorylate the 4S cluster in vitro. Further supporting a direct mechanism, GR co-IPs from BMMΦ and THP1 MΦ revealed dexamethasone-induced GRIP1:GR:CDK9 ternary complex formation (Fig. 2e).

To interrogate whether CDK9’s role as a GRIP1 kinase could be uncoupled from its role in transcriptional activation, we treated cells with triptolide, a global inhibitor of Pol II-mediated transcription. As expected, triptolide induced Pol II degradation^39^ and blocked activation of GR target genes in both THP1 MΦ and BMMΦ (Supplementary Fig. 5d). Nonetheless, dexamethasone-induced FVP-sensitive phosphorylation persisted (Supplementary Fig. 5d) indicating that GRIP1 phosphorylation by CDK9 can occur independent of transcriptional activation. Given this result and that GRIP1 phosphorylation requires a GR:GRIP1 interaction^24^, we propose that GR recruits CDK9 to modify GRIP1. Additional evidence in support of this model is presented below.

### Phosphorylation augments GRIP1 function as a GR coactivator

**Fig. 3:**
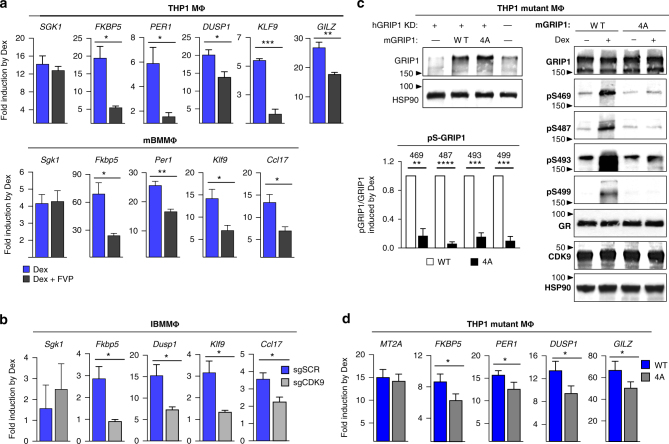
GRIP1 phosphorylation potentiates GR-mediated transcriptional activation. **a** THP1 cells or BMMΦ were treated ±dexamentasone (Dex)±50 nM flavopiridol (FVP) for 2 h and “fold induction” of indicated genes analyzed by RT-qPCR, normalized to β-actin and expressed relative to untreated or FVP alone (=1); mean+SEM are shown; *n* ≥ 3. **b** The induction by Dex (30 min) of GR target genes in single guide (sg)CDK9 and scrambled sgSCR control IBMMΦ was analyzed by RT-qPCR as in **a**; mean + SD is shown, *n* = 4. **c** THP1 cells transduced with SCR shRNA (-) or those depleted of endogenous hGRIP1 (+) from Fig. 1a, were stably transduced with WT or 4A mGRIP1 (see “Methods”), as indicated, and GRIP1 expression tested by immunoblotting with HSP90 is a loading control (left). WT- and S469A/S487A/S493A/S499A (4A)-expressing THP1 cells were treated ±Dex for 2 h and GRIP1 (total and phospho-isoforms), GR, CDK9 and HSP90 were visualized by immunoblotting (right). Quantification was performed as in Fig. 2c and expressed relative to WT (=1); mean + SEM are shown; *n* ≥ 3. Full-size western blots are shown in Supplementary Fig. 10g. **d** WT and 4A THP1 cells were treated ±Dex for 2 h and the expression of GR target genes was analyzed by RT-qPCR as in **a**; mean + SEM are shown, *n* ≥ 3. In each panel *P < 0.05, **P < 0.01, ***P < 0.001, ****P < 0.0001; unpaired, two-tailed Student’s *t*-test

To assess the functional consequences of GRIP1 phosphorylation, we disrupted either the function of CDK9 or the GRIP1 phosphosites and evaluated GR target gene expression. CDK9 inhibition by FVP attenuated dexamethasone induction of a subset of genes in both THP1 MΦ (*PER1, DUSP1, KLF9, GILZ*) and BMMΦ (*Fkbp5, Dusp1, Klf9, Ccl17*), while leaving the induction of *SGK1*/*Sgk1* unaffected (Fig. 3a), consistent with GRIP1 not participating in the activation of Sgk1 and ruling out a uniform effect of FVP on GR-mediated gene activation. Furthermore, the same genes affected by FVP in BMMΦ were sensitive to CDK9 depletion in IBMMΦ (Fig. 3b), corroborating a critical role of GRIP1 phosphorylation in GC induction of these genes in murine macrophages.

We next utilized our GRIP1-KD THP1 cells, which stably produce shRNA specific for hGRIP1 (Fig. 1a), to stably overexpress full-length murine WT or 4A GRIP1 via lentiviral delivery (Fig. 3c, left; Supplementary Fig. 6a). The inability of the mGRIP1 4A mutant to be phosphorylated in response to dexamethasone was confirmed by immunoblotting (Fig. 3c), with S487 phosphorylation being the best control as our anti-mouse pS487 antibody is species-specific and only recognizes exogenous mGRIP1 phosphorylation. Importantly, the loss of the 4S cluster in these mGRIP1 4A THP1-mutant MΦ resulted in attenuated induction of a subset of canonical and anti-inflammatory GR targets *(FKBP5, PER1, DUSP1, GILZ)* by dexamethasone, while *MT2A* was unaffected (Fig. 3d), consistent with its insensitivity to GRIP1 depletion (Supplementary Fig. 1c). As expected, the apparent effect of the 4A substitution was milder than that of kinase inhibition because overexpression of 4A GRIP1 should at least partially override its mutant phenotype.

Taken together, three different approaches in human and mouse macrophages point to an important regulatory function of GRIP1 phosphorylation in the GR-mediated activation of a specific panel of canonical and anti-inflammatory genes.

### GRIP1 phospho-isoforms occupy distinct GR-bound sites

**Fig. 4:**
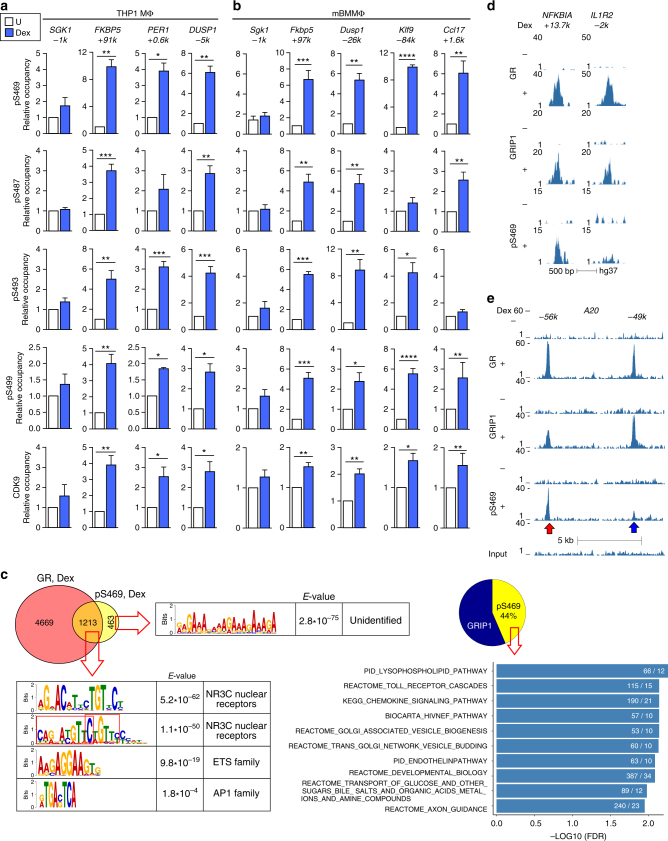
GRIP1 phospho-isoforms occupy GR target genes in a site-specific manner. THP1 cells (**a**) or BMMΦ (**b**) were incubated with dexamentasone (Dex) for 1 h, and the occupancy of phospho- (p)S469-, pS487-, pS493-, and pS499-GRIP1 as well as CDK9 was assessed at GRIP1-occupied sites from Fig. 1 by ChIP-qPCR as in Fig. 1f. Mean + SEM are shown (*n* ≥ 3, *P < 0.05, **P < 0.01, ***P < 0.001, ****P < 0.0001; unpaired, two-tailed Student’s *t*-test). **c** ChIP-seq for pS469-GRIP1 was performed in THP1 MΦ and Dex-induced peaks were called using MACS2 with pS469 peaks in untreated THP1 cells set as a background. (Left, top) Overlaps between pS469 and GR peaks in Dex-treated cells were determined as in Fig. 1c (Venn diagram), and the fraction of total GRIP1-bound sites that overlapped with pS469 peaks is shown (pie chart; see “Methods”). The union of peaks detected in two pS469-GRIP1 ChIP-seq experiments was used for the analysis (Supplementary Fig. 7). (Left, bottom) Ab initio sequence motif discovery and overrepresentation was assessed as in Fig. 1c. Red rectangles indicate overlapping NR3C half-sites. (Right) Gene-peak associations were analyzed using GREAT as described in “Methods”. **d** THP1 MΦ ChIP-seq read density profiles for genes showing GR and GRIP1 peaks with (*NFKBIA*) or without (*IL1R2*) S469 phosphorylation or **e** a pair of GR:GRIP1 binding sites nearby the *A20* locus both with (red arrow) and without (blue arrow) pS469

Our phosphosites of interest are within 30 aa of each other and may together form a novel GC-induced protein:protein interaction interface. Alternatively, distinct pGRIP1 isoforms with individual phospho-serines present in different combinations may occupy specific GBS. We explored these possibilities by ChIP-qPCR in THP1 MΦ and BMMΦ using phosphosite-specific antibodies and antibodies against CDK9. Consistent with the absence of GRIP1 at the *SGK1*/*Sgk1* GBS (Fig. 1c), neither pGRIP1 nor CDK9 was detected at this site in THP1 MΦ or BMMΦ (Fig. 4a, b) despite the expected dexamethasone-induced recruitment of CDK9 to the SGK1/Sgk1 TSS (as well as those of FKBP5/Fkbp5 and PER1/Per1) as part of transcription elongation machinery (Supplementary Fig. 6b). In contrast, at GBS with dexamethasone-dependent enrichment of GRIP1 (Fig. 1c—THP1: *FKBP5* +*91k, PER1* +*0.6k, DUSP1 -5k*; BMMΦ: *Fkbp5* +*97k, Dusp1 -26k, Klf9 -84k, Ccl17* +*1.6k*), GRIP1 was variably phosphorylated and CDK9 was co-recruited (Fig. 4a, b). Indeed, at the *PER1* GBS in THP1 MΦ and the *Klf9* GBS in BMMΦ, GRIP1 was phosphorylated at every serine except S487; similarly, at the Ccl17 GBS in BMMΦ, GRIP1 lacked S493 phosphorylation; whereas all four serines were modified at other GRIP1-occupied GBS tested. Thus, GRIP1 phosphorylation is not uniform, but may be acting as a code dictating distinct GRIP1 functions at specific genes.

To test how widespread GRIP1 phosphorylation is at the genomic level, we performed pS469-GRIP1 ChIP-seq in THP1 MΦ. Similar to total GRIP1 peaks in untreated cells, pS469 peaks in untreated cells showed minimal overlap between replicas, lower average read counts per peak than in dexamethasone-treated cells and an enrichment of low-complexity sequences in motif overrepresentation analysis (Supplementary Fig. 7a–b). Conversely, using ChIP-seq data from untreated cells as background (Supplementary Fig. 7c–h), we observed that roughly 3/4 of dexamethasone-induced pS469 peaks (1213 of 1676) overlapped with GR peaks and that GRIP1 was phosphorylated at this residue at 44% (996 of 2286 peaks) of its total binding sites (Fig. 4c, Supplementary Fig. 4c). Consistent with dexamethasone-induced total GRIP1 cistrome, among GRIP1:pS469-GRIP1-overlapping peaks, NR3C and ETS motifs were highly overrepresented and NR3C was centrally enriched (Fig. 4c, Supplementary Fig. 7e, f). Similar to GRIP1 cistrome, genes associated with GRIP1:pS469-GRIP1-overlapping peaks belonged to immune-related pathways (Fig. 4c, right panel). We next evaluated read distributions and confirmed pS469-GRIP1 peaks at *FKBP5* +91k, *PER1* +0.6k, *DUSP1* −5k, and *KLF9* +17k (Supplementary Fig. 7g). Strikingly, we identified additional GBS associated with anti-inflammatory targets at which GRIP1 was S469-phosphorylated (*NFKBIA* +13.7k), unphosphorylated (*IL1R2* -2k), or exhibited differential phosphorylation at GBS clusters (at least two sites within 5 Kb from each other) related to the same gene (*A20* −49k vs. −56k) (Fig. 4d, e). In fact, clusters of GR:GRIP1 binding sites often displayed differential S469 phosphorylation: see *FAM53B*, *RDX*, and *ST14* (Supplementary Fig. 7h).

Given that CDK9 co-occupied GBS with pGRIP1, we speculated that activated GR recruits CDK9 to modify GRIP1 and that CDK9 recruitment in response to dexamethasone persists even in the absence of GRIP1. Indeed, in GRIP1-depleted (shGRIP1) THP1 MΦ or GRIP1 KO BMMΦ the dexamethasone-induced enrichment of GR and CDK9 was unchanged despite abrogated GRIP1 occupancy (Supplementary Fig. 8a–c).

### Phosphorylation affects GRIP1 occupancy and function at GBS

**Fig. 5:**
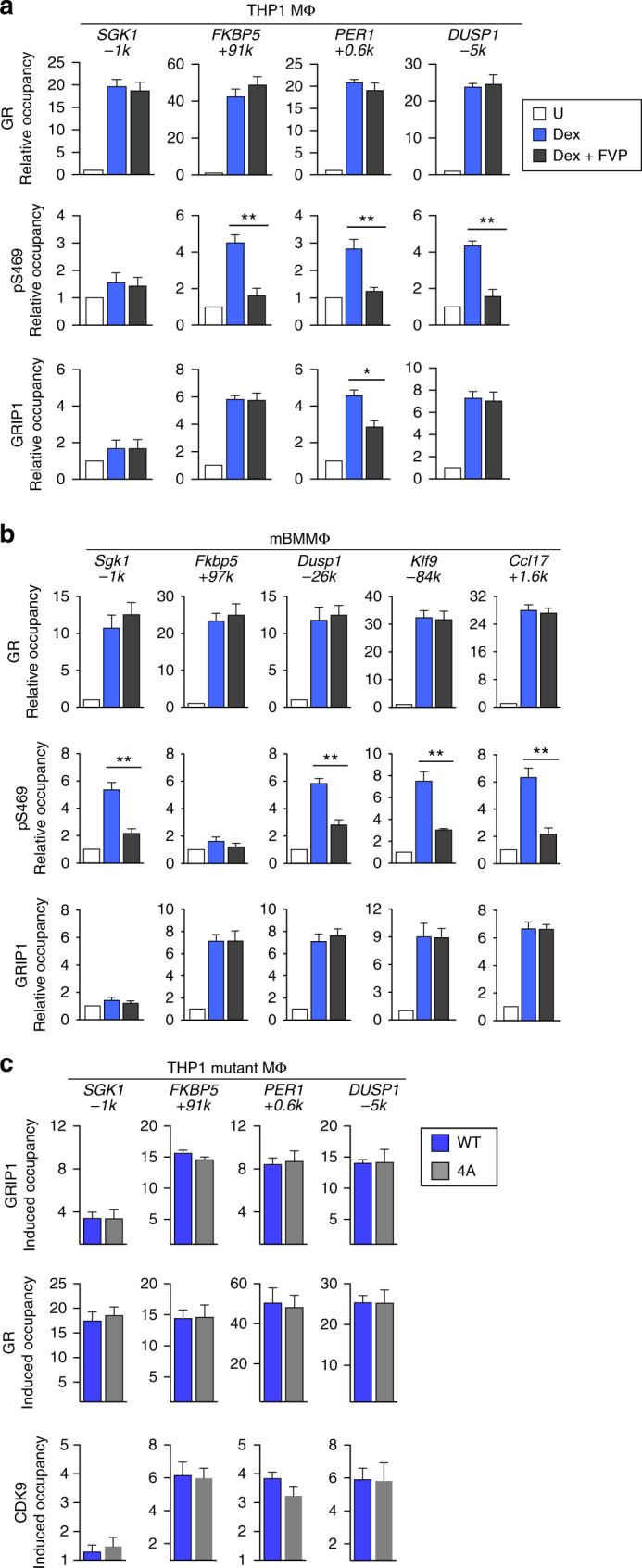
GRIP1 phosphorylation affects its localization at a subset of genomic sites. THP1 cells (**a**) and BMMΦ (**b**) were treated ±dexamentasone (Dex) ±50 nM flavopiridol (FVP) for 1 h, and the occupancy of GR, pS469-GRIP1 and GRIP1 was analyzed by ChIP-qPCR as in Fig. 1f. **c** THP1-mutant-MΦ expressing WT or S469A/S487A/S493A/S499A (4A) mGRIP1 were treated ±Dex for 1 h and the expression of indicated genes was analyzed by RT-qPCR as in Fig. 1a and expressed relative to untreated cells for each genotype (=1). Means + SEM are shown (*n* ≥ 3, *P < 0.05, **P < 0.01; one-way ANOVA with Bonferroni’s multiple comparison test)

In principle, phosphorylation may affect multiple GRIP1 functions including its stability, subcellular localization, recruitment to genomic binding sites and/or, ultimately, interactions with additional components of GR complexes or basal transcriptional machinery. Given that GRIP1 levels are unchanged by dexamethasone and FVP that alter GRIP1 phosphorylation status (Fig. 2) and that GRIP1 is a constitutively nuclear protein^40–43^, we tested whether GRIP1 phosphorylation is important for its localization to GBS. Comparing dexamethasone-treated MΦ ±FVP, we found that FVP did not affect GR recruitment but, as expected, dramatically reduced occupancy of pS469-GRIP1 in both THP1 MΦ and BMMΦ (Fig. 5a, b, top and middle). Interestingly, loss of phosphorylation correlated with a reduction of GRIP1 occupancy at the *PER1* GBS in THP1 MΦ (Fig. 5a, bottom), suggesting that phosphorylation contributed to GRIP1 targeting or retention at this GBS. Conversely, GRIP1 occupancy at the *FKBP5* or *DUSP1* GBS in THP1 cells or the *Fkbp5, Dusp1, Klf9*, or *Ccl17* GBS in BMMΦ was unaffected by FVP. Consistent with inhibitor data, dexamethasone-induced enrichment of WT and 4A mGRIP1 as well as CDK9 and GR, were indistinguishable between THP1-mutant MΦ lines (Fig. 5c). Given that loss of phosphorylation impaired the ability of GRIP1 to serve as a GR coactivator for these genes (Fig. 3), we envision a scenario in which phosphorylation of the serines generates novel surfaces for protein:protein interactions and facilitates events downstream of GRIP1 loading (for example, recruitment of secondary cofactors or communication with basal machinery).

### GRIP1 corepressor function is phosphorylation independent

**Fig. 6:**
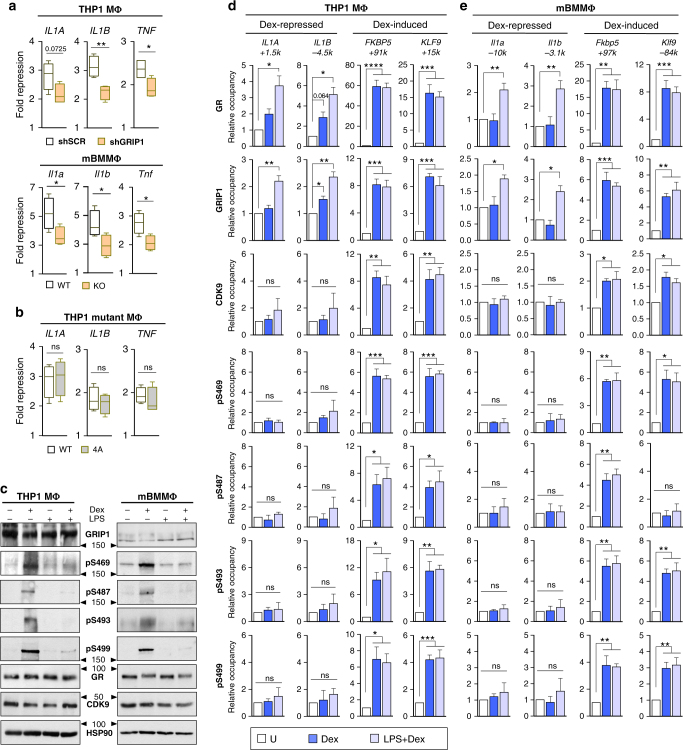
GRIP1 phosphorylation does not potentiate its GR corepressor function. **a** THP1 cells (small hairpin scrambled (shSCR) or shGRIP1) or BMMΦ (WT or GRIP1 KO) were treated ± 10 ng ml^−1^ LPS and ± Dex for 1 h and the expression of indicated genes was analyzed by RT-qPCR as described in Fig. 1a. “Fold repression” was defined as the quotient of the transcript level at lipopolysaccharide (LPS) over LPS+dexamethasone (Dex) condition and is shown as Tukey box-and-whisker plots (*n* = 4). **b** THP1 cells expressing WT or S469A/S487A/S493A/S499A (4A) mGRIP1 were treated as in (a) and fold repression of indicated genes is shown. ns, non-significant. **c** THP1 MΦ or BMMΦ were treated ± Dex ± LPS for 2 h, as indicated, and the levels of GRIP1, GRIP1 phospho-isoforms, GR, CDK9 and HSP90 were evaluated by immunoblotting (quantified in Supplementary Fig. 9d; full-size blots are shown in Supplementary Fig. 10h). THP1 MΦ (**d**) or BMMΦ (**e**) treated with ±Dex ±LPS for 1 h were analyzed for GR, GRIP1, CDK9 and phospho-GRIP1 occupancy by ChIP-qPCR as in Fig. 1f (see Supplementary Fig. 9e for more genes). Shown are mean + SD, *n* ≥ 3, *P < 0.05, **P < 0.01, ***P < 0.001, **** *P* < 0.0001; one-way ANOVA with Dunnett’s multiple comparison test

GRIP1 has emerged as an important GR corepressor at “tethering” complexes, in which GR is recruited to chromatin through protein:protein interactions with DNA-bound AP1 or NF-κB and represses their target genes (such as *Tnf, Il1a*, and *Il1b*)^20–22^. Given that repression of hundreds of pro-inflammatory genes by the liganded GR is a critical component of the therapeutic actions of GCs, we first confirmed the role of GRIP1 in glucocorticoid repression of lipopolysaccharide (LPS, a TLR4 agonist)-activated genes in THP1 MΦ or GRIP1 KO BMMΦ from the LysM-Cre*GRIP1* ^*fl/fl*^ mice, in which the contribution of GRIP1 to GR repression has not been evaluated. Consistent with our published results^20,22^, dexamethasone repression of LPS-induced *IL1A/Il1a, IL1B/Il1b*, and *TNF/Tnf* was attenuated in GRIP1-deficient macrophages of both species relative to their GRIP1-sufficient counterparts (Fig. 6a). Importantly, GRIP1 depletion did not affect the ability of LPS to induce these genes (Supplementary Fig. 9a), demonstrating that the difference in “fold repression” arises from attenuated function of GR.

To corroborate the anti-inflammatory function of GRIP1 in the LysM-Cre*GRIP1* ^*fl/fl*^ mice in vivo, we challenged the GRIP1 KO and matching WT mice with LPS (5 mg kg^−1^ intraperitoneally) and assessed the hallmarks of septic shock syndrome. Consistent with our published studies^23^, GRIP1-deficient animals displayed significantly higher mortality (*P < 0.05; Kaplan–Meier analysis and the log-rank test) and lost a greater amount of body weight than WT as early as 24 h post injection (Supplementary Fig. 9b). To evaluate gene expression changes in macrophages at the site of 12-h LPS exposure, we performed peritoneal gavages and sorted peritoneal macrophages (CD45^+^CD11b^+^; CD11c^−^CD3^−^B220^−^Gr1^−^; see Supplementary Fig. 9c for gating strategy). In line with in vitro results, relative expression of GR-sensitive pro-inflammatory cytokines *Tnf*, *Il1a*, and *Il1b* was greatly elevated, whereas that of GC-stimulated anti-inflammatory *Ccl17* and, to a lesser extent, *Klf9* was reduced in the GRIP1 KO macrophages compared to WT (Supplementary Fig. 9c). Interestingly, the anti-inflammatory gene *Dusp1* known to be strongly induced by inflammatory signaling and additively by GCs^7^, was expressed at higher levels in the KO macrophages, consistent with their exaggerated inflammatory response. Importantly, the apparent attenuation of GC regulation in GRIP1 KO in vivo occurred despite significantly (*P* < 0.05; unpaired, two-tailed Student’s *t*-test) elevated levels of circulating endogenous GC corticosterone at 12 h post injection, corroborating the inability of these animals to control systemic inflammation (Supplementary Fig. 9b).

**Fig. 7:**
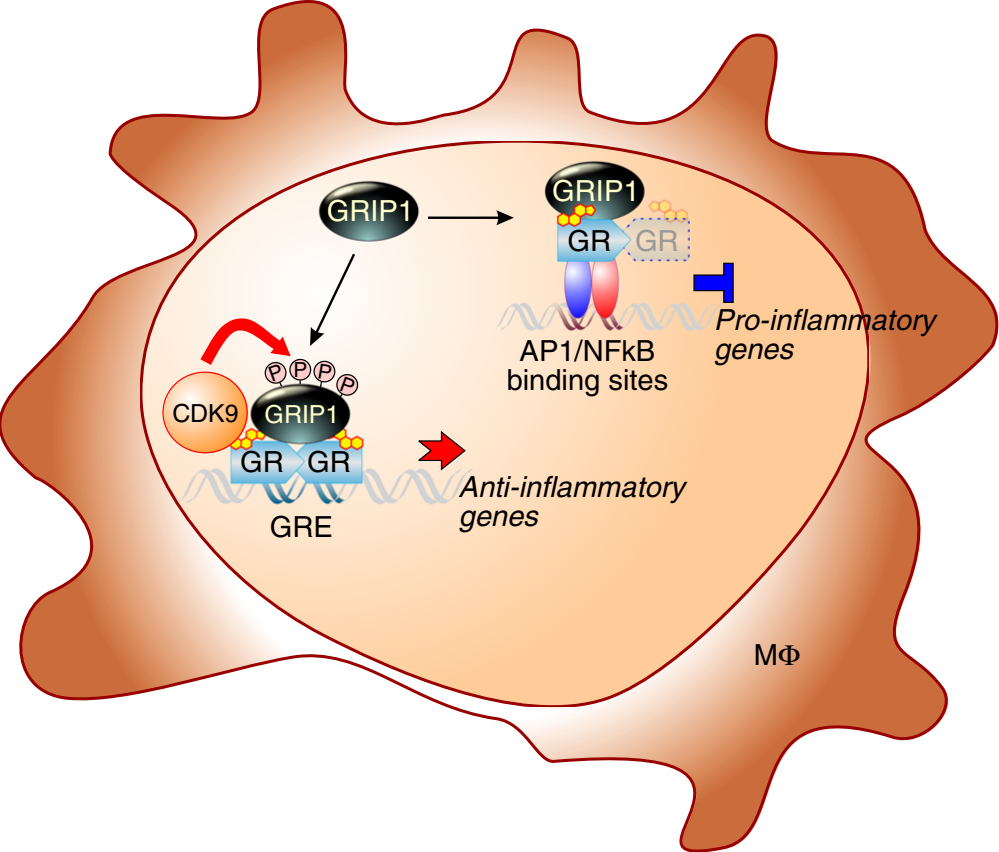
Differential utilization of GRIP1 phosphorylation at GR-regulated genes. Diagramed is a macrophage (MΦ) with GR bound at a palindromic GC response element (GRE) activating transcription of anti-inflammatory genes or “tethered” to AP1 or NF-κB repressing pro-inflammatory genes. GRIP1 is recruited to both types of complexes but undergoes phosphorylation by CDK9 at the palindromic GRE only

To test whether the 4S phosphorylation cluster was important for GRIP1 corepressor function, we compared GC repression of LPS-induced *IL1A, IL1B*, and *TNF* in THP1-mutant MΦ expressing WT or 4A mGRIP1 and found it to be indistinguishable (Fig. 6b). Given the apparent lack of a role for target serines in GRIP1 corepression, we assessed whether they were still phosphorylated under LPS+Dex repressing conditions. Unexpectedly, LPS greatly attenuated dexamethasone-induced GRIP1 phosphorylation at each serine in THP1 MΦ and BMMΦ (Fig. 6c; quantified in Supplementary Fig. 9d), raising the possibility that LPS treatment globally attenuated GRIP1 phospho-isoform occupancy and GR-mediated induction of genes that are pGRIP1-dependent. We investigated this scenario by comparing the dexamethasone-induced occupancy of GR, GRIP1, CDK9, and pGRIP1 at GR activation or repression binding sites ±LPS exposure. As expected from the “tethering” nature of GR repression complexes at κB sites, GR and GRIP1 were recruited to *Il1a* and *Il1b* in BMMΦ in the presence of dexamethasone+LPS only and not dexamethasone alone (Fig. 6e). Similarly, in THP1 MΦ (Fig. 6d), GR and GRIP1 recruitment to *IL1A* and *IL1B* κB sites was most apparent upon Dex+LPS co-treatment, despite a modest enrichment of GR and GRIP1 at the *IL1B* κB site with Dex alone, likely due to residual p65 and AP1 presence after phorbol myristate acetate-induced differentiation^44,45^. Interestingly, however, neither GRIP1 phospho-isoforms nor CDK9 were detected at κB sites of *IL1A/Il1a* or *IL1B*/*Il1b* (Fig. 6d, e), consistent with pGRIP1 not participating in GR repression complexes. In contrast, at GR-activated genes *FKBP/Fkbp5*, *KLF9/Klf9*, *PER1*, and *Ccl17* the Dex-induced occupancy of GR, GRIP1, CDK9 and all GRIP1 phospho-isoforms was fully resistant to LPS (Fig. 6d, e and Supplementary Fig. 9e), suggesting that pGRIP1 can persist at many GR-induced genes despite the general reduction of GRIP1 phosphorylation (Fig. 6c). Accordingly, dexamentasone-dependent, FVP-sensitive activation of these genes was similarly unaffected by LPS (Supplementary Fig. 9f, g). Together, these data indicate GRIP1 phosphorylation in macrophages specifically facilitates coactivator properties, but is dispensable for GRIP1 corepressor functions (Fig. 7).

## Discussion

Transcriptional cofactors coordinate and stabilize large multi-protein complexes, integrate diverse upstream signals and, ultimately, transduce information to basal transcriptional machinery and chromatin. The p160s have emerged as critical cofactors of NRs, which represent an important class of therapeutic targets. Despite a growing appreciation of the importance of p160s from gene ablation studies, specific NR:p160 relationships in relevant contexts that underlie their KO phenotypes are missing. Here we show a non-redundant role of the p160 family member GRIP1 in the GR activation of a panel of canonical and anti-inflammatory genes in mouse and human macrophages. Indeed, other cofactors, including the other two well-expressed p160s, failed to compensate for GRIP1 loss, even under conditions of stable GRIP1 deletion.

Genome-wide analyses of GRIP1 distribution yielded two unexpected observations. First, our genomic analysis in macrophages of both species argues that under liganded conditions, GRIP1 is a dedicated GR cofactor. This commitment is supported by the predominance of GREs and other sequence motifs known to be targeted by GR in overlapping peaks and the dramatic dexamethasone-induced expansion of GRIP1 cistromes, which substantially overlap GR cistromes; this was in striking agreement with recent data on single-molecule imaging of GR:GRIP1 association with chromatin^46^. The second surprising finding was an overrepresentation of immune-related pathways associated with GR:GRIP1 binding sites in GC-treated macrophages. This result did not reflect GR:GRIP1 co-localization at tethering “transrepression” sites as our macrophages were not exposed to inflammatory triggers that promote AP1 or NF-κB mobilization, which is a prerequisite for the formation of GR tethering complexes. Indeed, no p65 motifs were enriched in any of our GRIP1 cistromes; instead, sites of GR:GRIP1 co-occupancy after dexamethasone exposure were dominated by palindromic GREs typically associated with direct GR-inducible targets. It is therefore likely that the immune signature of the overlapping cistromes comprises anti-inflammatory genes activated by GR, including Dusp1, Klf9, Il10, A20, or Ccl17. Combined, these data suggest that increased sensitivity to sepsis observed in our macrophage-specific GRIP1 KO and that of previously described GR KO mice^22,47^ in part stems from suboptimal GC activation of anti-inflammatory genes, in accord with several earlier reports^5,47,48^.

Given that GRIP1 has been implicated in multiple physiological processes, including reproduction, metabolism, and circadian clock^31,49–51^, it would be informative to assess the genomic relationships of GRIP1 and other transcription factors, including NRs, in different contexts. Currently, GRIP1 cistromes are few: genome-wide occupancy of hGRIP1 has only been described in the MCF7 breast cancer line^52^; in murine cells, GRIP1 cistromes were reported in cardiomyocytes, prostate and liver tissues^50,53,54^. Its distribution in macrophages under conditions of acute GC stimulation has not been previously evaluated, although certain levels of co-occupancy with GR have been reported in anti-inflammatory (“M2”) BMMΦ generated via long-term GC-driven polarization followed by a 3-h exposure to LPS^55^. Given a wealth of recent studies on transcriptionally and epigenetically distinct states of macrophage activation^56–58^, future assessment of the role of GRIP1 in macrophage programming represents an extremely fruitful direction to pursue.

Mechanisms underlying specificity in cofactor utilization remain largely unresolved. With over 300 proteins reportedly participating in GR signaling^4^, it is reasonable to speculate that “more selective” non-GRIP1 components may dictate its recruitment and function. Here we describe GR-driven mobilization of CDK9 and GRIP1 phosphorylation on the 4S cluster (S469, S487, S493, and S499) as a more refined mechanism by which GR directs its own coregulator to activation complexes associated with a subset of canonical and anti-inflammatory genes (Fig. 7). Conceivably, differential GRIP1 phosphorylation imparts a deeper level of specificity to its action. Indeed, our analysis revealed that pS469-GRIP1 occupied 44% of total GRIP1 binding sites and that different isoforms existed in GR:GRIP1 complexes within the same cluster of GBS. A comprehensive mapping of GRIP1 phospho-isoform binding, as ChIP-seq quality antibodies to individual sites become available, would be a promising avenue for future studies. It is critical to acknowledge, however, that this approach is constrained by the properties of phospho-specific antibodies, epitope accessibility and local chromatin features to a much greater extent than transcription factor binding analysis.

Mechanistically, GRIP1 phosphorylation may have multiple non-mutually exclusive outcomes. We showed that loss of phosphorylation attenuated the ability of GRIP1 to localize to a specific GBS. Another possibility is that the 4pS cluster (or less phosphorylated variants thereof) represents a novel protein-binding interface. Phosphorylation is a powerful modulator of protein:protein interactions: phosphorylation of transcriptional cofactors CARM1 and LSD1 was recently shown to even bypass a requirement for ligand in gene activation by Estrogen Receptor-α^59^. In fact, phosphorylation of GR itself creates a new surface permissive for binding of certain coregulators while blocking others^60^. Interrogation of this mechanism via proteomic approaches holds the promise of uncovering important modulators of GR:GRIP1 actions in macrophages.

This is the first report describing CDK9 occupancy at an NR response element and its involvement in factor-specific transcriptional regulation. CDK9 is a key component of the Pol II elongation machinery, which makes its functions in more specific transcriptional pathways difficult to assess. Our data argue that detection of CDK9 at the GREs is not a “spill-over” from the TSS due to spatial proximity. Indeed, no CDK9 was detected at the *SGK1* GRE at −1 kb despite pronounced GC-induced CDK9 recruitment to the promoter 1 kb away. A scenario wherein, instead, GR recruits CDK9 to modify GRIP1 at a GBS is corroborated by the observation of GR:GRIP1:CDK9 ternary complexes in GC-treated macrophages. We note that this interaction was only detectable after dual crosslinking and could potentially be DNA-dependent. Whether phosphorylation precedes or follows GR:GRIP1 DNA binding is not directly assessable in our experimental system. However, in light of the specificity of pGRIP1 recruitment even in the context of the same gene, our findings are consistent with an established model in which the GRE serves as an allosteric ligand for GR, shaping its structure and, thus, specifying cofactor composition. Given that phosphorylation strictly depends on the GR:GRIP1 interaction, we posit that DNA, even if not required for phosphorylation per se, greatly enhances the stability of the GR:GRIP1 complex and thereby increases substrate availability for CDK9. We also do not exclude the possibility that additional GRIP1 kinases target the 4S cluster in a combinatorial or inter-dependent manner whereby CDK9 phosphorylates a specific serine, which primes phosphorylation of an adjacent serine by another kinase. CDK9 itself is increasingly recognized as a potential drug target in cancers, AIDS, and is being tested in mouse models of arthritis^61^, highlighting the importance of better understanding its diverse functions.

Intriguingly, GRIP1 phosphorylation is restricted to GR activation complexes with no apparent contribution to GRIP1 corepressor functions in the gene panel tested (Fig. 7). Because GC and TLR signaling are mutually antagonistic, we considered the possibility that LPS-induced pathways globally inhibit GRIP1 phosphorylation, perhaps by imparting modifications to GRIP1 precluding phosphorylation or upregulating a putative GRIP1 phosphatase. Unexpectedly, and despite a decrease of whole-cell GRIP1 phosphorylation, the occupancy of pGRIP1 and CDK9 specifically at the GBS associated with dexamethasone-activated genes and their induction were fully resistant to LPS. How this selectivity is accomplished remains to be explored, however, given relatively low levels of GRIP1 in macrophages, signal-induced phosphorylation could serve as a mechanism that enables GRIP1 to engage in mutually complementary anti-inflammatory actions at both GR-activated and GR-repressed genes at the expense of genes whose glucocorticoid regulation is not essential in inflammatory contexts.

NR coregulators are emerging as potential therapeutic targets due to the striking phenotypes of their deletion and documented deregulation in disease (for review, see ref. ^62^). Targeting NR coregulators in future therapeutics will likely add to the arsenal of molecules that affect NR signaling pathways, which comprise over 20% of current pharmaceuticals, with GCs being the most commonly prescribed class of drugs worldwide^63^. A non-redundant, phosphorylation-specified function of GRIP1 as GR coactivator complements its role as a GR corepressor^7,20,22^ in the context of macrophages and inflammation and may represent an important avenue toward achieving drug selectivity.

## Methods

### Mice

C57BL/6 mice (National Cancer Institute, Charles River Laboratories; strain code 556) and their transgenic derivatives were maintained in the Hospital for Special Surgery and Weill Cornell Barrier Animal Facility in full compliance with the protocol approved by the Institutional Animal Care and Use Committee. Homozygous WT (wt/wt*GRIP1* ^*fl/fl*^) and GRIP1 KO (LysM-Cre*GRIP1* ^*fl/fl*^) mice were generated previously^31^.

GRIP1 KO and WT mice were injected intraperitoneally with 5 mg kg^−1^ of LPS (Sigma) and monitored for 36 h. Mice were weighed at the time of injection and 24 h later.

For sorting of peritoneal macrophages, animals were euthanized via CO2 inhalation after 12 h of 5 mg kg^−1^ LPS i.p. administration and peritoneal gavages with PBS performed. Antibody staining for flow cytometry included: CD45-PerCP-C5.5 (anti-mouse CD45, clone 30-F11), Cd11b-PE (anti-mouse/human CD11b, clone M1/70), CD3-FITC (anti-mouse CD3e, clone 145-2C11), CD11c-FITC (anti-mouse CD11c, clone N418), Gr1-FITC (anti-mouse Ly-6G/Ly-6C (Gr1), clone RB6-8C5), B220-FITC (anti-mouse/human CD45R/B220) (Biolegend; 1:100 dilution each).

Serum GC levels were determined using the Corticosterone ELISA kit (Abcam, ab108821) and as per manufacturer’s directions. Serum was collected from the blood of euthanized mice12 h after 5 mg kg^−1^ LPS i.p. injection.

### Cell culture and treatments

THP1 cells (ATCC TIB-202) were maintained in RPMI-1640 (Invitrogen) supplemented with 10% fetal bovine serum (FBS; Atlanta Biologicals) and 5–10 nM β-mercaptoethanol (Sigma). THP1 cells were differentiated by 25 ng ml^−1^ phorbol myristate acetate (Sigma) for 18–24 h followed by a 48 h to obtain THP1 MΦ. Small hairpin (sh)GRIP1 and scrambled shSCR cell lines were generated via lentiviral knockdown as described below. mGRIP1 WT and S469A/S487A/S493A/S499A (4A) were generated via lentiviral overexpression in the shGRIP1 parental line as described below.

Peripheral blood mononuclear cells were obtained from leukocyte preparations of healthy volunteers (New York Blood Center) and separated by Ficoll (Invitrogen) density gradient centrifugation. CD14^+^ human monocytes were purified from PBMC using magnetic anti-CD14 beads (Miltenyi). Positively selected monocytes were cultured in RPMI-1640 with 10% FBS, l-glutamine (Invitrogen), penicillin/streptomycin (P/S; Invitrogen) and 20 ng ml^−1^ recombinant human macrophage colony stimulating factor (Peprotech) for 72 h to obtain monocyte-derived hMΦ.

BMMΦ were prepared from 8- to 10-week-old male mice as described^22^. In brief, tibia and femur bone marrow was flushed and incubated in 1 g l^−1^ glucose-containing DMEM (Invitrogen) supplemented with 20% FBS and 20% L-Cell conditioned media for 5 days. Adherent cells were then scraped and plated at 2 × 10^7^ in 150 mm plates in DMEM—20% FBS to be treated the following day.

IBMMΦ were generated previously via stable introduction of Raf and Myc^64^ and maintained in DMEM containing 1 g l^−1^ glucose supplemented with 10% FBS and P/S. Single guide (sg)CDK9 and scrambled sgSCR cell lines were generated via lentiviral knockdown as described below.

For cell treatments, 100 nM dexamethasone (Sigma) and 10 ng ml^−1^ LPS (Sigma) were used. For kinase inhibitor experiments, the indicated doses of flavopiridol (FVP) (to 50–100 nM final) were added to cell culture media 30 min before subsequent treatments. For global transcription blockade, triptolide (Abcam) was added to a final concentration of 10 μM 15 min prior to subsequent treatments.

### Lentiviral knockdown and overexpression

HEK 293T cells (ATCC) were prepared at 75–90% confluency in Opti-MEM (Invitrogen) supplemented with GlutaMAX (Invitrogen), Sodium Pyruvate (Invitrogen) and 5% FBS and transfected using Lipofectamine 3000 and P3000 reagent (Invitrogen) with the packaging plasmids psPAX2 (Addgene) and pMD2.G (Addgene) and one of the following plasmids: shGRIP1 plasmid, shSCR control, WT mGRIP1, 4A mGRIP1, sgCDK9, or sgSCR control. Six hours post transfection, media was removed and replaced with fresh media containing P/S. Viral media was collected 30 h and 52 h post transfection. Lentivirus was concentrated using Centricon-70 Plus Centrifugal filters (Millipore) and added to THP1 or IBMMΦ cells at an MOI of 50:1 in the presence of 5 μg ml^−1^ polybrene (Sigma). Cells were positively selected by antibiotic resistance [200 ng ml^−1^ puromycin (Sigma) or 300 μg ml^−1^ geneticin/G418 (Invitrogen) for 1–2 week] or by cell sorting at the Weill Cornell Flow Cytometry Core on a FACS Aria II using fluorometric markers—mCherry, mAmetrine or TagBFP.

### Immunoblotting and protein immunoprecipitations

Whole cell extracts were prepared using standard procedure in RIPA buffer (10 mM Tris-HCl pH 8.0, 1 mM EDTA, 0.5 mM EGTA, 140 mM NaCl, 5% glycerol, 0.1% Na deoxycholate, 0.1% SDS, 1% Triton X-100). Nuclear extracts were prepared using NE-PER Nuclear and Cytoplasmic Extraction Reagents (Thermo) following manufacturer’s instructions. The following inhibitors were used: Protease Inhibitor Cocktail powder (Sigma, P2714), Phosphatase Inhibitor Cocktail 3 (Sigma, P0044), 1 mM phenylmethylsulfonylfluoride, 1 mM sodium orthovanadate, and 1 mM sodium fluoride. Commercial antibodies were used against GRIP1 (Abcam, ab10491, 1:2000 and BD, 611319, 1:500), GR (Santa Cruz, sc-1004x, 1:1000), CDK9 (Santa Cruz, sc-8338x, 1:1000), HSP90 (Cell Signaling, 4874 S, 1:2000) and GST (Abcam, ab1956, 1:500). Immunoblotting with phosphosite-specific antisera was performed at 1:3000, except for anti-pS469 that was used at 1:500. Precision Plus Protein Dual Color Standards (Biorad) were used to identify molecular weights. Blots shown are representative of ≥3 independent experiments. Densitometric analysis of immunoblots was performed using ImageJ (NIH).

For IPs, cells were double cross-linked with 2 mM disuccinimidyl glutarate (DSG, Proteochem) for 30 min at RT followed by 1% methanol-free formaldehyde (Thermo) for 10 min at RT. The reaction was quenched by 0.125 M glycine for 5 min. Cells were then washed with PBS, scraped and lysed in RIPA buffer + inhibitors. GR IPs were performed using mouse anti-GR (Santa Cruz, G-5, sc-393232) at 1:100 with 40 μl of pre-washed Dynabeads Protein A (Novex, Thermo) O/N at 4 °C and were washed 5× in RIPA buffer. IP samples and inputs were de-crosslinked at 95 °C for 5–10 min. Immunoblotting was performed using rabbit polyclonal antibodies as described above.

### In vitro kinase assays

Recombinant WT and 4A GST–GRIP1_322–631_ fragments were produced in *E. coli* and purified as described^23^ and cyclin T1-CDK9 was produced in baculovirus. Assays were performed in Tris-Cl (pH 7.6), 5 mM DTT, 5 mM MgCl_2_ with 10 μCi of [γ^32^P]-ATP (6000 Ci mM ^−1^) or 40 μM ATP. Visualization was performed by immunoblotting as described above or by autoradiography and Coomassie blue staining.

### RNA isolation and RT-qPCR

Total RNA was isolated from cells with the RNeasy Plus Mini Kit (Qiagen), subjected to random-primed cDNA synthesis, and gene expression analyzed by qPCR with Maxima Sybr Green/ROX/2x master mix (Fermentas) on StepOne Plus real-time PCR system (Applied Biosystems) using standard protocols and the δδCt method. Primers are listed in Supplementary Table 1.

### ChIP and ChIP-seq

THP1 MΦ, hMϕ, and BMMΦ were double-crosslinked and prepared for sonication as described previously^31^. Cross-linked chromatin was sonicated using a Bioruptor Pico (Diagenode) for THP1 MΦ and hMΦ or an S220 Ultrasonicator (Covaris) for BMMΦ to generate DNA fragments ∼150–600 bp. Inputs were taken from cleared lysates and the rest were incubated for 4 h at 4 °C with the following antibodies to precipitate protein:DNA complexes: GR (Santa Cruz, M20x, sc-1004x or N499), GRIP1 (Abcam, ab10491 or Bethyl A300-346A), phospho-specific GRIP1 antibodies, or CDK9 (Santa Cruz, H169x, sc-8338x). An aliquot of 40–50 μl of pre-washed Dynabeads Protein A were added per IP and incubated O/N at 4 °C. Beads were washed 8 times in modified RIPA buffer (50 mM HEPES [pH 7.6], 100 mM LiCl, 1 mM EDTA, 1% NP-40, 0.7% Na-deoxycholate) once with TE with sodium chloride (10 mM Tris pH 8.0 and 1 mM EDTA, 50 mM NaCl). Each reaction was then incubated in TE-0.5% SDS-200 μg ml^−1^ proteinase K for 2 h at 55 °C, followed by 6 h at 65 °C to reverse cross-links. DNA was purified with Qiagen’s PCR purification kit following manufacturer’s directions. Real-time qPCR primers are listed in Supplementary Table 1. For GR, GRIP1 and pGRIP1 ChIP-seq, 10–30 ng of IP or input material was used. The amount and sonication quality of DNA was tested with Qubit (Thermo) and Bionalyzer 2100 (Agilent Technologies) before library construction using Illumina TruSeq ChIP-Seq Library Prep Kit and sequencing by a HiSeq 2500 system (four to six samples/SR lane, 50 bp reads). Library preparation and sequencing was performed by the Weill Cornell Epigenomics Core.

### Plasmid generation

Human-specific shRNAs to GRIP1 were cloned into pMK1221 (Addgene #84220), an RNA Pol II-driven lentivirus shRNA vector. The ability of individual shRNAs to knock-down GRIP1 in NALM-6 cells (ATCC CRL-3273) after lentiviral transduction was assessed by western blot^65^. The following shRNA KD GRIP1 worked most efficiently and was used: GRIP1 Sense Strand Sequence: TAGACCTAATTTGTAGACTTAA

Mouse WT and 4A full-length GRIP1 constructs were created from mouse cDNA stepwise using the QuikChange II Site Directed Mutagenesis Kit (Agilent). Full-length murine WT and 4A were BFP-tagged at the N-Terminus through cloning into the pTag-BFP-C vector (Evrogen) and PCR-cloned using Q5 High-Fidelity DNA Polymerase (NEB) and T4 DNA ligase (NEB) in-frame into the pWPI vector (Addgene) using the following primers:

CDS Start + PmeI: 5′-TAAGCAGTTTAAACATGAGCGAGCTGATTAAGGAG-3′

CDS End + PmeI: 5′-TGCTTAGTTTAAACTCAGCAGTATTTCCGAGATGC- 3′

sgRNA that targeted CDK9 or no specific target (sgSCR) were cloned into the all-in-one lentiCRISPR v2 vector (Addgene), in which the selection marker puromycin was replaced by the fluorescent protein mAmetrine.

All constructs were confirmed by sequencing.

### Statistical tests

Sample sets were compared by unpaired, two-tailed Student’s *t*-test with *P* < 0.05 set as a significance threshold when only two samples were being compared except in Supplementary Fig. 9c where *P* < 0.1 was set as a threshold.

One-way ANOVA with Dunnett’s multiple comparison test was used when two or more samples were being compared to a control sample. One-Way ANOVA with Bonferroni’s multiple comparison test was used when three or more samples were being compared with two or more crosswise comparisons. *P* < 0.05 was set as a significance threshold.

Mann–Whitney test was used when data distribution deviated from normal. *P* < 0.05 set as a significance threshold.

Survival analysis was performed using Kaplan–Meier analysis and the log-rank test with *P* < 0.05 as a significance cutoff.

### ChIP-seq statistical analysis

Sequencing quality control was performed using *FASTQC* and adapters, when needed, were trimmed using *trimmomatic*. 50 bp single-end reads were aligned to the human genome (GRCh37/hg19) or mouse genome (mm10) with *bowtie2*. Aligned BAM files were converted into bigwig format for data visualization purposes. GR peaks in BMMΦ were called using the Hotelling Observer signal filtering algorithm implemented in CLC BIO genomics workbench with *P* < 0.01 as a significance threshold^66^. Peak calling for all other data sets was performed with *MACS2* with the following parameters:

for GR in THP1 MΦ: --format = BAM --gsize 2451960000 --bw = 300 ---slocal 1000 --llocal 10000 --keep-dup 1 -qvalue 0.01;

for GRIP1 in BMMΦ: --format = BAM --gsize 2150570000 --bw = 200 --to-large --ratio 1.0 --slocal 1000 --llocal 10000 --keep-dup 1 –pvalue 0.0001;

for GRIP1 in THP1 MΦ --format = BAM --gsize 2451960000 --bw = 200 --ratio 1.0 --slocal 3000 --llocal 15000 --keep-dup 1 --pvalue 1e-05

with a matching input file to estimate background read distribution. pS469 peaks in dexamethasone-treated samples were called vs. pS469 untreated samples to only obtain dexamethasone-induced peaks with the following parameters: --format = BAM --gsize 2451960000 --bw = 200 --to-large --ratio 1.0 --slocal 1000 --llocal 100000 --keep-dup 1 --pvalue 0.0001.

All called peaks were filtered against ENCODE blacklisted human and mouse regions with empirically high mappability^67^.

Consensus GR peaks were determined using *subsetByOverlaps* function from R *GenomicRanges* package and were used for further set analyses with the minimum overlap of 1 nt and visualized with *makeVennDiagram* function from *ChIPpeakAnno* package.

Because GRIP1 peak sets from individual GRIP1 replicas form larger overlaps with GR peak set than between each other, we defined GRIP1 union peaks set as following: (GR GRIP1_rep1 ∩ GR) U (GR GRIP1_rep2 ∩ GR) (Supplementary Figs. 3d and 4d) and used this subset for all further peak set analyses.

Distributions of MACS2 pileup values in different subsets of GR peaks were compared using kernel density estimation from R *sm* package as previously described^31^.

Peak annotation relative to known genomics features was performed using *ChIPpeakAnno* package^68^ (R, Bioconductor) with *TxDb.Mmusculus.UCSC.mm10.knownGene* annotation package that is based on the mm10 genome UCSC knownGene table for BMMΦ and *TxDb.Hsapiens.UCSC.hg19.knownGene* based on the GRCh37/hg19 USCS known Gene table for THP1 MΦ.

The functional annotation of peak-associated genes was performed using the R interface (https://github.com/jokergoo/rGREAT) for GREAT^69^, the whole genome as a background and the “basal plus extension” gene association rule with the following parameters: adv_upstream = 5.0kb, adv_downstream = 1.0 kb, adv_span = 100 kb, MSigDB (Broad Institute) was used as a source of reference for functional gene sets. The significance of gene set enrichment was evaluated using FDR-adjusted hypergeometric test and the top 15 enriched gene sets were selected.

Ab initio analysis of overrepresented sequences in ChIP-seq peaks was performed using MEME-ChIP suite with *MEME*, *DREME* to determine overrepresented sequences and *CentriMO* to determine sequences with positional bias. *E*-values estimate the expected number of motifs in an experimental set of sequences compared to random sequences of the similar size. Sequencing motifs with *E-*values under 0.0001 were considered significant.

### Data availability

All sequencing data have been deposited into GEO database under the accession number GSE99887. All relevant data are available from the authors.

## Electronic supplementary material

Supplementary Information
