# Supplementary material for: Glucocorticoid-induced phosphorylation by CDK9 modulates the coactivator functions of transcriptional cofactor GRIP1 in macrophages
Source: Nat Commun. 2017 Nov 23;8:1739. doi: 10.1038/s41467-017-01569-2 (PMC5700924; doi:10.1038/s41467-017-01569-2)
Supplement: Supplementary file 1 — Supplementary Information [file 41467_2017_1569_MOESM1_ESM.pdf]

Supplementary Figure 1

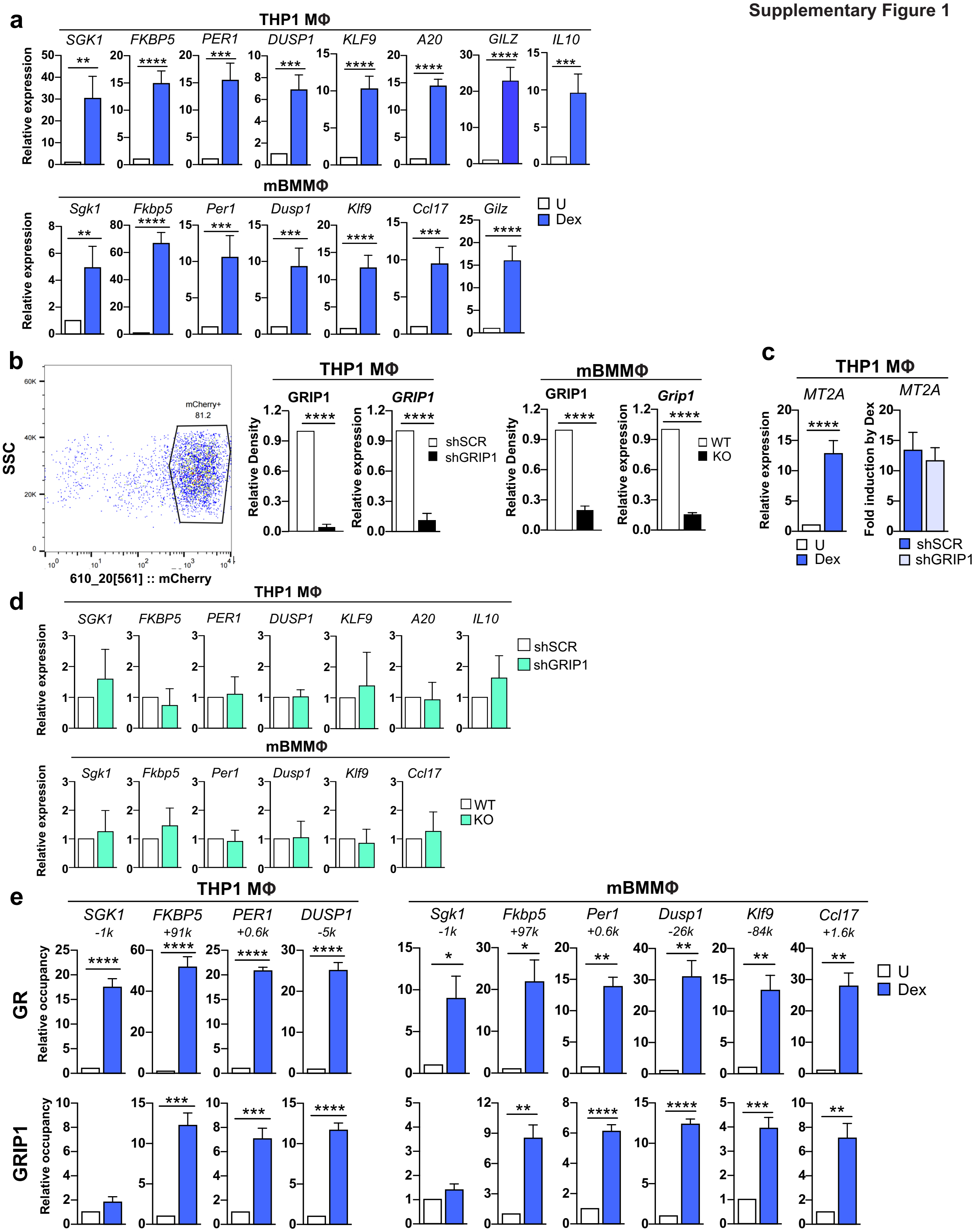

Supplementary Fig. 1 | GR and GRIP1 cooperate in GC-mediated gene induction in macrophages.
(**a**) The induction of GR target genes in THP1 MΦ or mouse BMMΦ following a 2-h dexamethasone (Dex) treatment was analyzed via RT-qPCR as described in Fig. 1a. Shown are mean+SD and n=3. (**b**) Transduced viable THP1 cells (small-hairpin (sh)GRIP1 and scrambled shSCR) were selected by flow cytometry based on lack of Dapi staining (viable) and positive for mCherry expression (left). GRIP1 protein depletion in Fig. 1a-b was quantified using densitometry relative to control (=1; shSCR or WT) and GRIP1 RNA depletion assessed by RT-qPCR, normalized to β-actin and expressed relative to non-depleted cells (=1); shown are mean+SD, n=3. (**c**) Parental (left) or shSCR- and shGRIP1-transduced (right) THP1 cells were untreated (U) or treated with Dex for 2 h and MT2A transcript measured by RT-qPCR as described in Fig. 1a; shown are mean+SD, n=3. (**d**) Basal expression of indicated genes in THP1 MΦ or BMMΦ from Fig. 1a-b was measured by RT-qPCR as described in Fig. 1a; shown are mean+SD; n=3. (**e**) GR and GRIP1 recruitment to ChIP-seq sites shown in Fig. 1c-d was assessed by ChIP-qPCR as described in Fig. 1f. In each panel, shown are mean+SEM (n=3, * $p<.05$, ** $p<.01$, *** $p<.001$, **** $p<.0001$; unpaired, two-tailed Student's t-test).

Supplementary Figure 2

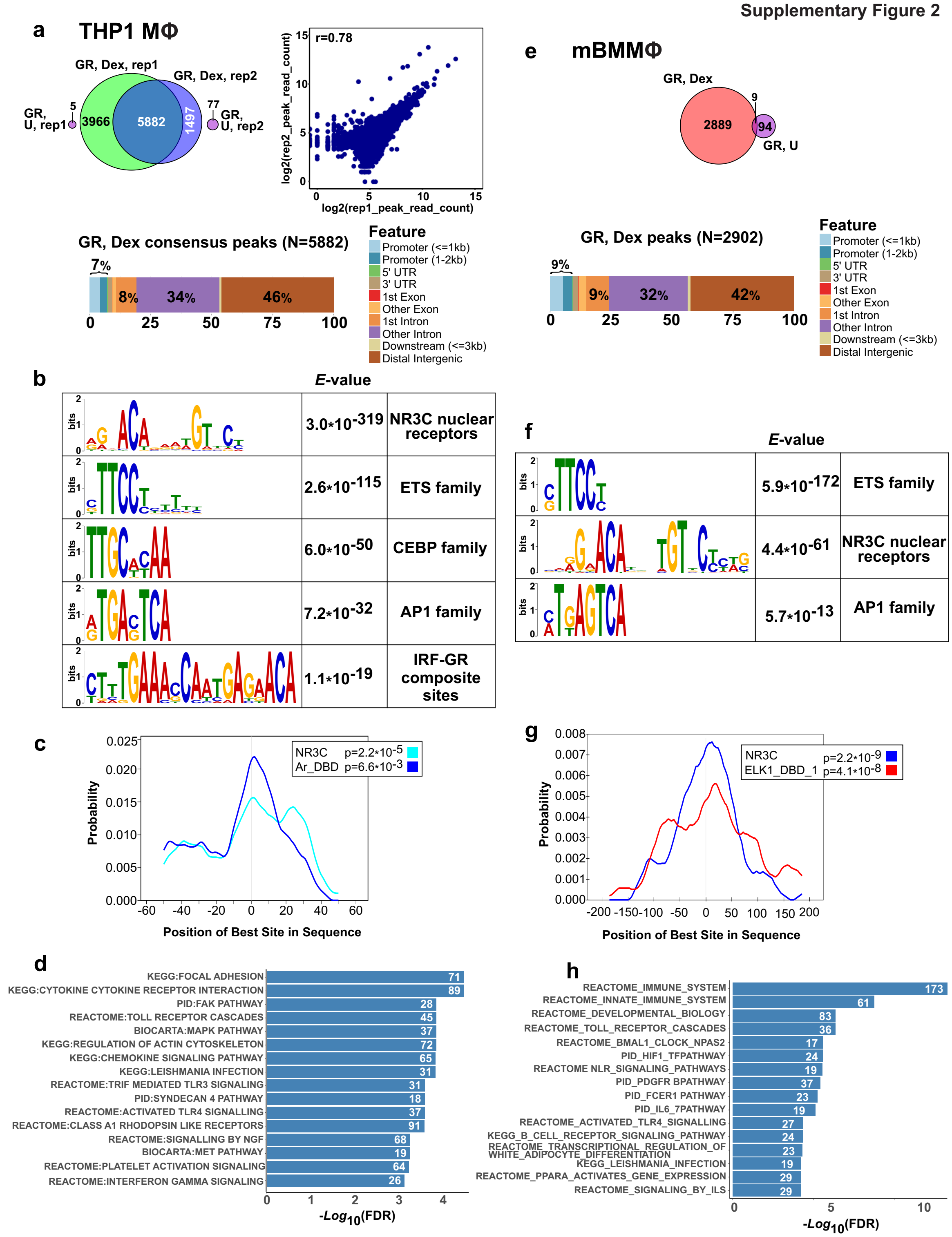

Supplementary Fig. 2 | GR ChIP-seq in THP1 MΦ and mouse BMMΦ.
(**a** and **e**) GR cistromes in untreated (U) or dexamethasone (Dex)-treated THP1 MΦ (**a** - top) and BMMΦ (**e** - top), and the distribution of GR peaks relative to known genomic features (**a** - bottom and **e** - bottom). The results of two replicates (rep) in THP1 MΦ and a single experiment in BMMΦ are shown. Peaks were called with MACS2 (**a**) and CLC Bio genomics workbench (**e**) with the FDR-corrected p-value for peaks detection in comparison to input DNA was set to 0.01. GR consensus peak set (**a**, left) was created by determining genomics interval overlaps between two replicates with *subsetByOverlap* function from GenomicRanges package (Bioconductor). To determine the concordance between replicas, an interval union of peaks called in each replica was used to calculate read numbers in each peak. Log-transformed read counts were plotted against each other and Pearson correlation between replicates was calculated (**a**, right). (**b** and **f**) *Ab initio* sequence motif discovery and overrepresentation of GR peak sets in THP1 MΦ (**b**) and BMMΦ (**f**) were performed as in Fig. 1c. (**c** and **g**) The centrality enrichment analysis of binding motifs identified by *ab initio* prediction with MEME/DREME in THP1 MΦ (**c**) and BMMΦ (**b**) was performed using CentriMo program of MEME suite. (**d** and **h**) Gene-peak associations in THP1 MΦ (**d**) and BMMΦ (**h**) was determined using GREAT. "Basal plus extension" peakgene association rule was used with the basal regulatory region set to -5000 - +1000 bases relative to annotated TSS that was further extended in both directions to the nearest gene's basal domain up to 100 kb.

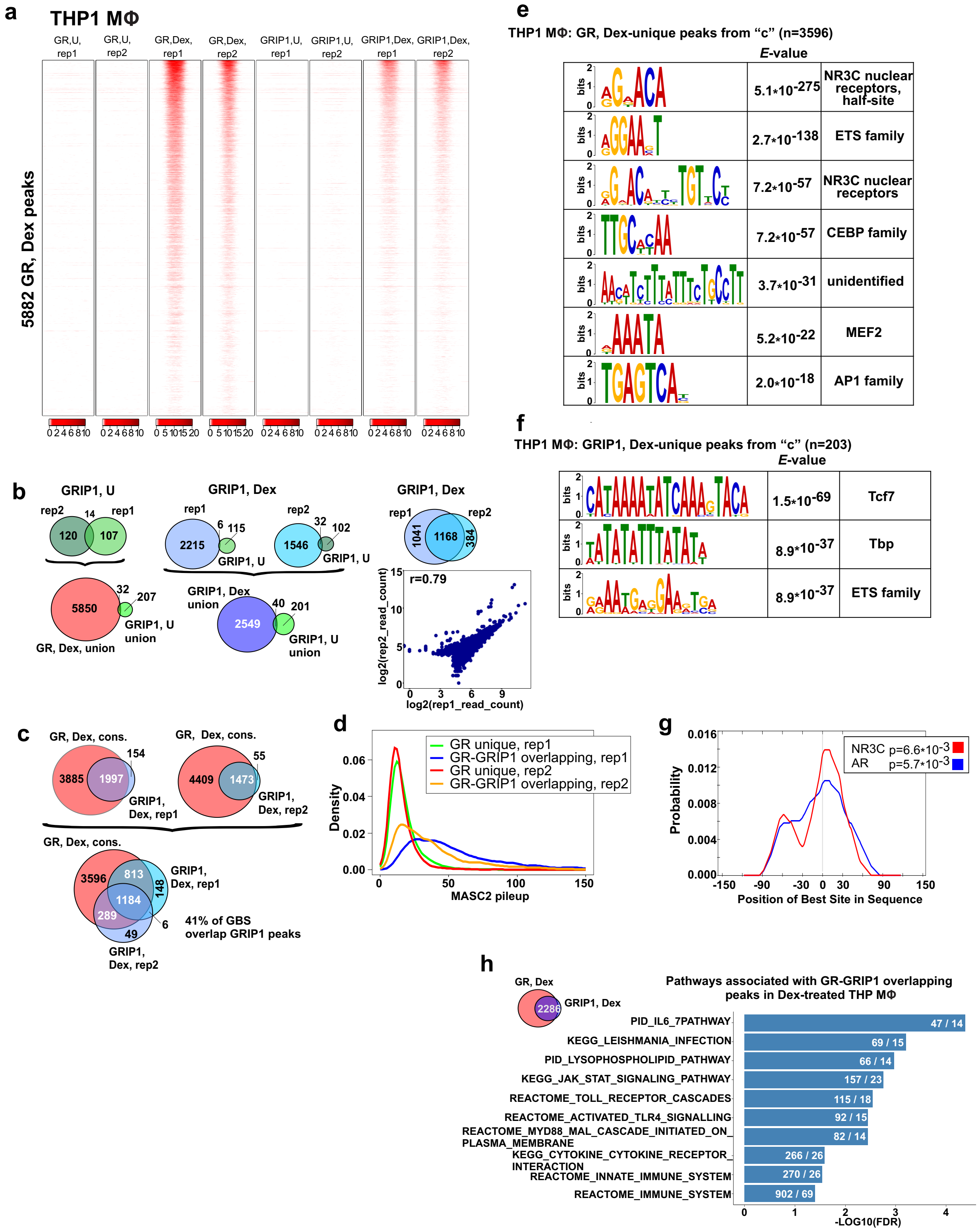

Supplementary Fig. 3 | The analysis of THP1 MΦ GRIP1 cistromes.
(**a**) Read distributions within 5882 Dex-induced GR consensus peaks (from Supplementary Fig. 2a) sorted by MACS2 pileup value in GR, dexamethasone (Dex), replicate (rep)1 sample. All identified GR peaks were extended by 150 bp at 3' and 5' ends, split into 100 bins, per bin density was calculated for all ChIP-seq experiments, normalized to the library size and plotted. (**b**) GRIP1 peaks were called with MACS2 with nominal p=0.00001 as a significance threshold. Set analysis of GRIP1 peaks in untreated (U) vs. Dex-treated THP1 cells indicates little overlap in GRIP1 binding. The concordance between replicas ("b", bottom right) was determined as in Supplementary Fig. 2a. (**c**) More Dex-induced GRIP1 peaks in replicate experiments overlap with GR peaks than between each other (b, top right). (**d**) Distribution of MACS2 pileup values in GR peak subsets indicate larger peak size in GR-GRIP1overlapping peak sets for both replicas. Probability density functions for MACS2 pileup values were calculated using nonparametric density estimations implemented in R sm package. *Ab initio* discovery of overrepresented sequence motifs in (**e**) GR-unique peaks in Dex treated- and (**f**) GRIP1-unique peaks in untreated THP1 MΦ. (**g**) The centrality enrichment analysis of binding motifs in GR-GRIP1 overlapping peaks in Dex-treated THP1 MΦ using CentriMo program of MEME suite. (**h**) *Ab initio* discovery of overrepresented sequence motifs in GRIP1-unique peaks in Dex-treated THP1 MΦ (**i**) Gene-peak associations in Dex-treated THP1 MΦ were analyzed with GREAT as in Supplementary Fig. 2h.

Supplementary Figure 4

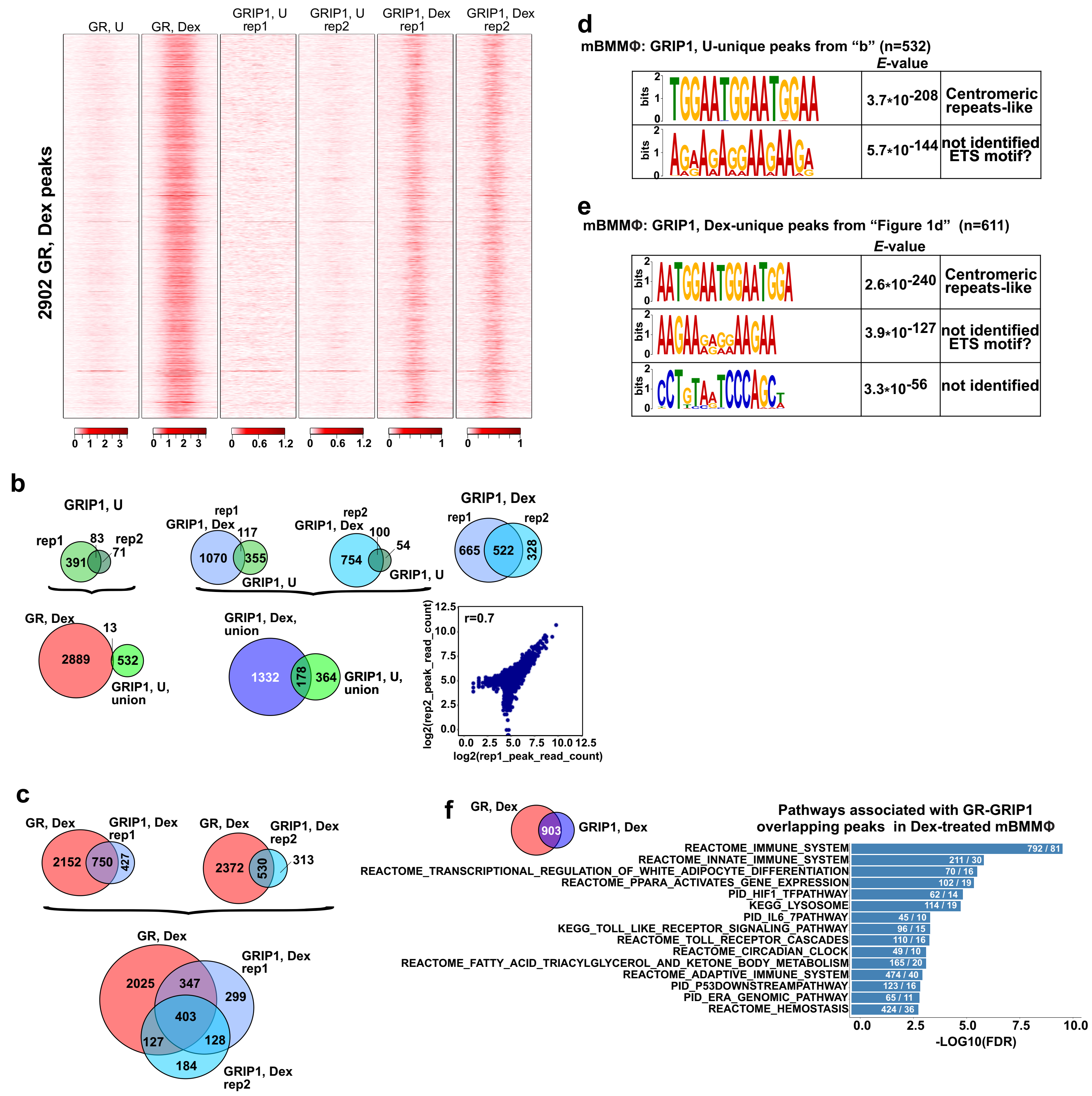

Supplementary Fig. 4 | The analysis of BMMΦ GRIP1 cistromes.
(**a**) Read distributions within 2902 GR peaks identified in dexamethasone (Dex)-treated BMMΦ. The analysis was performed as in Supplementary Fig. 3a. U, untreated. rep, replicate. (**b**) GRIP1 peaks were called using MACS2 with nominal p=0.00001 as a significance threshold and the set analysis of GRIP1 peaks in untreated and Dex-treated BMMΦ was performed as in Supplementary Fig. 3b. The concordance between replicas (b, bottom right) was determined as in Supplementary Fig. 2a and 3b. (**c**) More Dex-induced GRIP1 peaks for replicate experiments overlap with GR peaks than between each other. Ab initio discovery of overrepresented sequence motifs in unique GRIP1 peaks in (**d**) untreated and (**e**) Dex-treated BMMΦ. (**f**) Gene-peak associations in Dex-treated THP1 MΦ was analyzed with GREAT as in Supplementary Fig. 2h.

Supplementary Figure 5

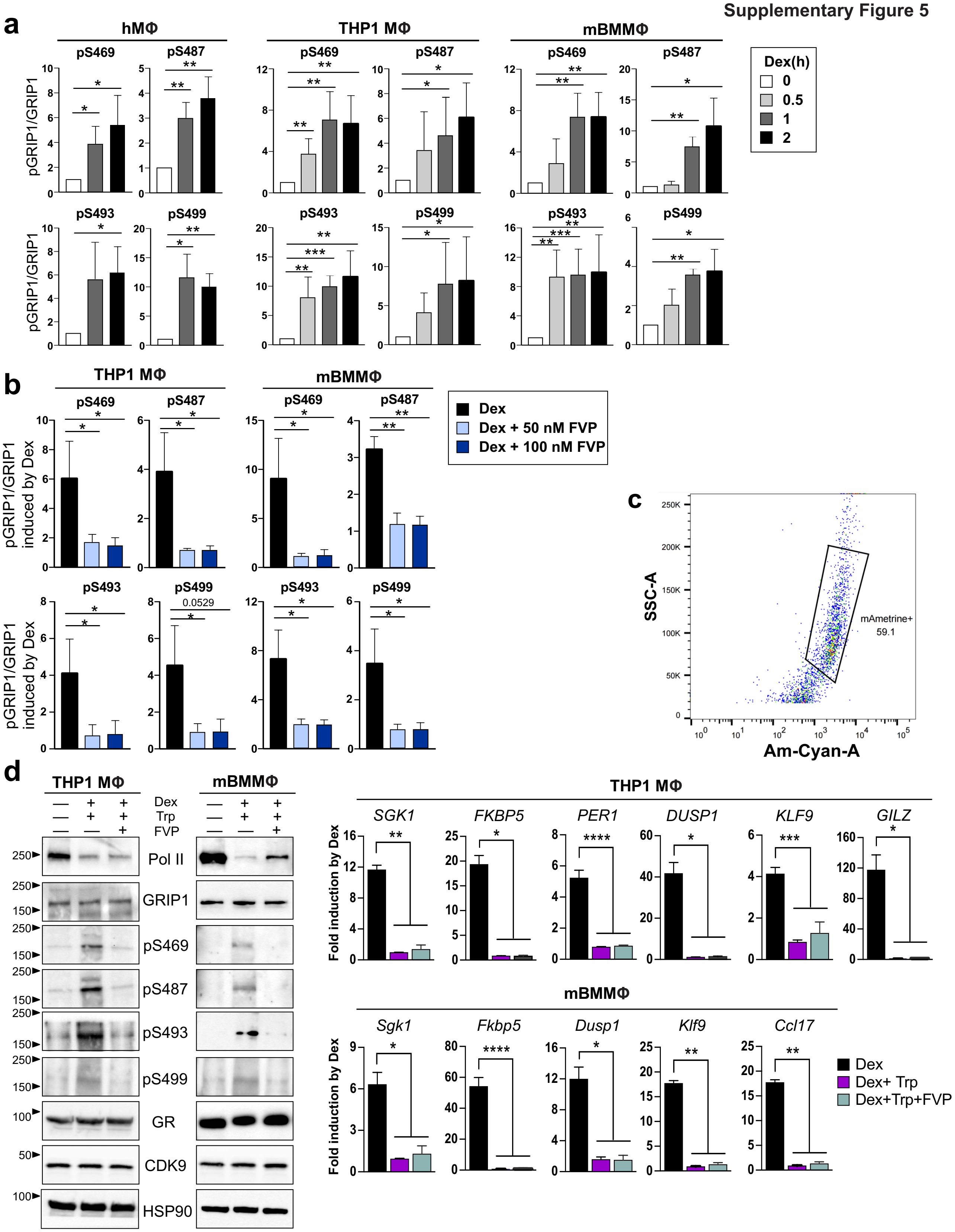

Supplementary Fig. 5 | Dex induces phosphorylation of GRIP1 by CDK9.
Densitometric analysis of immunoblot images from (**a**) hMΦ, THP1 MΦ and mouse BMMΦ treated with dexamethasone (Dex) in Fig. 2a or from (**b**) THP1 MΦ and BMMΦ treated with Dex and flavopiridol (FVP) was performed using phospho (p)GRIP1:GRIP1 ratios relative to untreated (=1). (**c**) viable Dapi staining-resistant positively-transduced single guide (sg)CDK9 and scrambled sgSCR lBMMΦ were selected on the basis of mAmetrine expression by flow cytometry. (**d**) THP1 MΦ or BMMΦ were treated with indicated combinations of Dex, Triptolide (Trp) and FVP, and the levels of GRIP1 (total or phosphorylated at S469, S487, S493 and S499), GR, CDK9 and HSP90 loading control assessed by immunoblotting (left) or the induction of indicated genes was analyzed by RT-qPCR as in Fig. 1e with untreated or Trp alone treatment as a control (=1, right).Full-size western blots are shown in Supplementary Fig. 10f. In each panel, mean+SEM are shown (n=3; * p<.05, ** p<.01, *** p<.001, **** p<.0001; One-Way ANOVA with Dunnett's multiple comparison test).

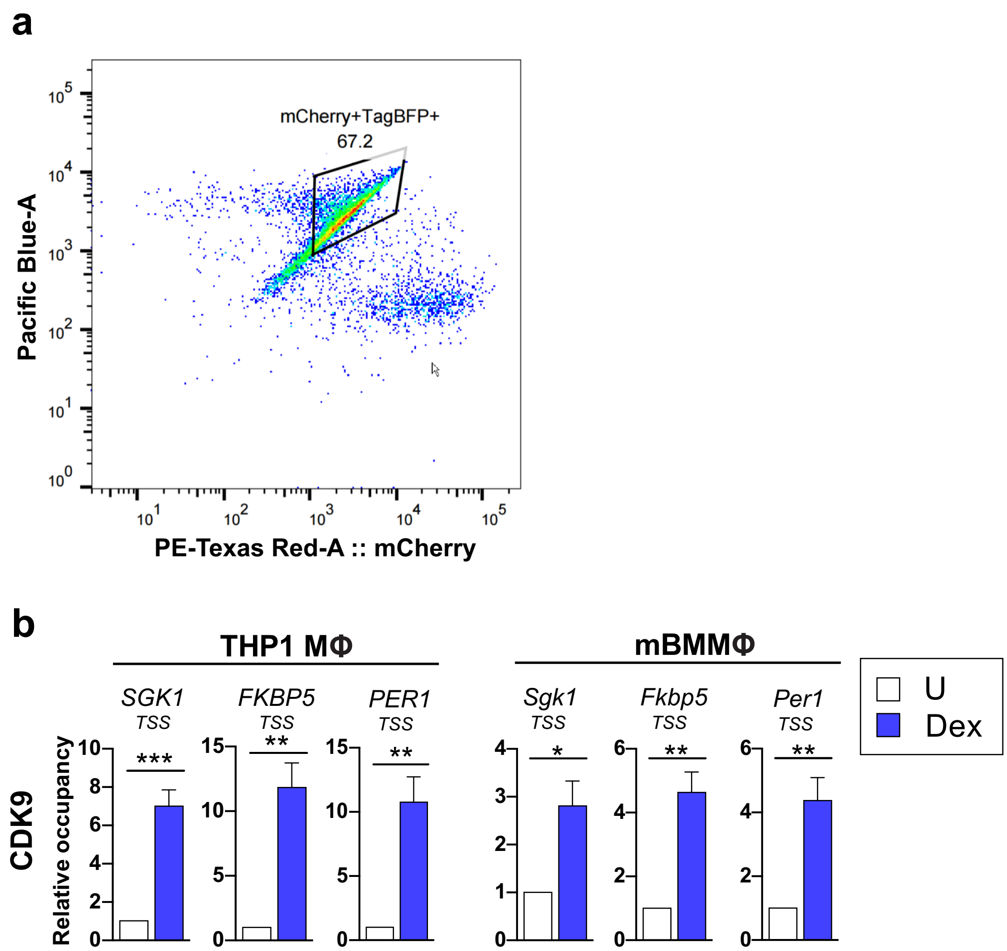

Supplementary Fig. 6 | Dexamethasone (Dex)-induced CDK9 recruitment to the transcription start site (TSS) of GR target genes.

(**a**) Small hairpin (sh)GRIP1 cells that were viable (negative for 7-AAD staining) and positively transduced with human-specific small hairpin (sh)GRIP1 and murine WT or S469A/S487A/S493A/S499A (4A) mGRIP1 plasmids were selected based on mCherry and TagBFP expression by flow cytometry. (**b**) Dex-induced CDK9 occupancy of the TSS of *SGK1/Sgk1*, *FKBP5/Fkbp5*, and *PER1/Per1* in THP1 MΦ or BMMΦ was assessed by ChIP-qPCR as in Fig. 4a-b. U, untreated. Mean+SEM are shown (n=4; * p<.05, ** p<.01, *** p<.001; unpaired two-tailed Student's t-test).

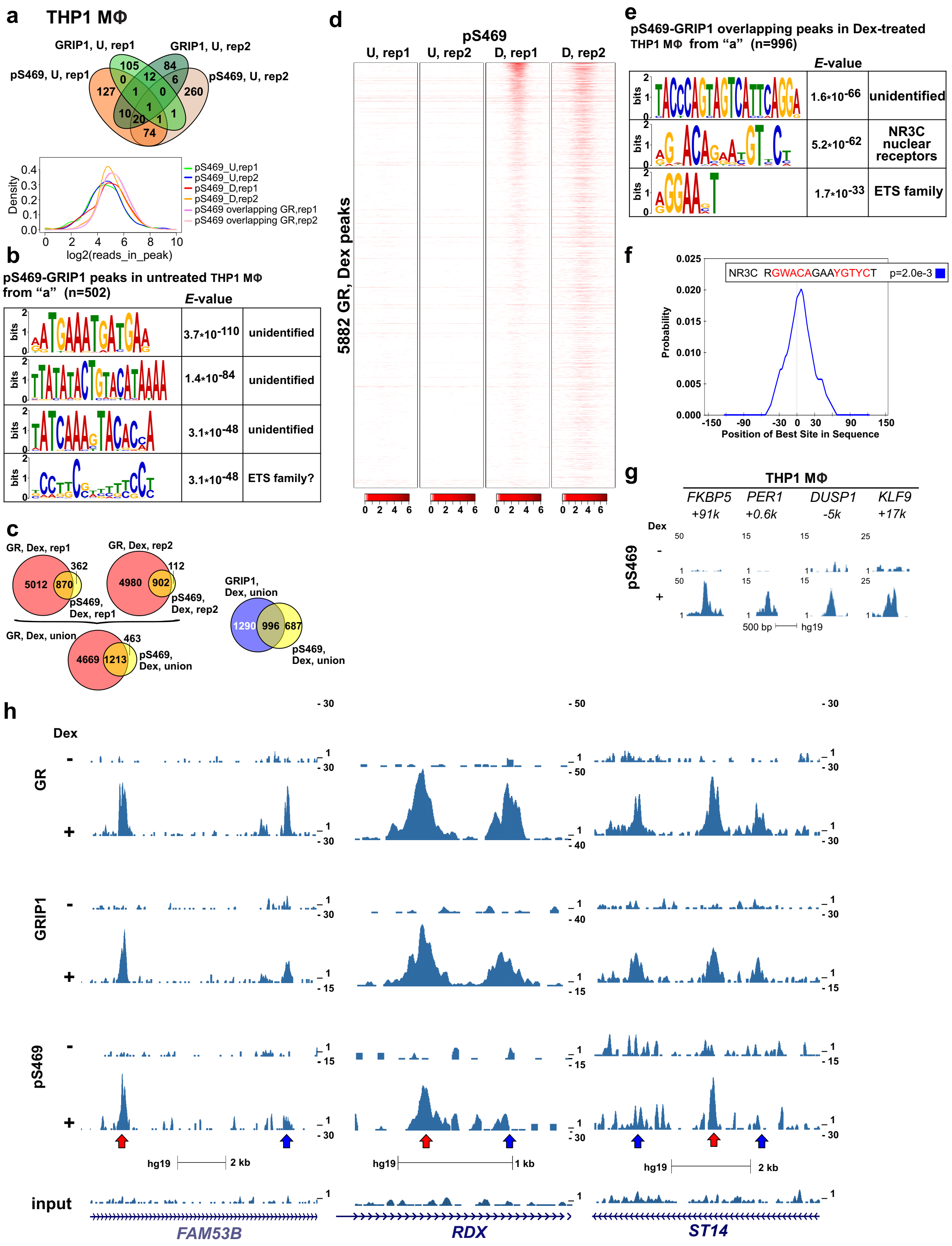

Supplementary Fig. 7 | GRIP1 is S469-phosphorylated in a GBS-specific manner in THP1 MΦ.
(**a**) Set analysis of GRIP1-pS469 and total GRIP1 peaks identified in untreated THP1 MΦ by MACS2 (top) and density plot of log-transformed read-in-peaks distribution for various subsets of pS469-GRIP1 peaks. U, untreated; D, dexamethasone-treated. (**b**) *Ab initio* discovery of overrepresented motifs in pS469-GRIP1 peaks from untreated THP1 MΦ. (**c**) Set analysis of pS469 peaks identified in dexamethasone (Dex)-treated THP1 MΦ by MACS2 with the p-value detection threshold of 0.0001. rep, replicate. (**d**) Read distributions in pS469 ChIP-seq samples within 5882 Dex-induced GR consensus peaks (from Supplementary Fig. 2a). U, untreated. (**e**) *Ab initio* discovery of overrepresented sequence motifs in GRIP1-pS469 overlapping peaks in Dex-treated THP1 MΦ indicates an overrepresentation of NR3C-binding motifs with (**f**) central enrichment. (**g**) pS469 ChIP-seq read distribution shows recruitment of pS469-GRIP1 to the sites analyzed by ChIP-qPCR in Fig. 1a. (**h**) Read distributions of GR, GRIP1 and pS469 at GR:GRIP1 clusters (n≥2 GR:GRIP1-bound sites within 10 Kb of each other) associated with *FAM53B*, *RDX* and *ST14* genes. The absence or presence of corresponding pS469 peaks is indicated by blue and red arrows, respectively.

Supplementary Figure 8

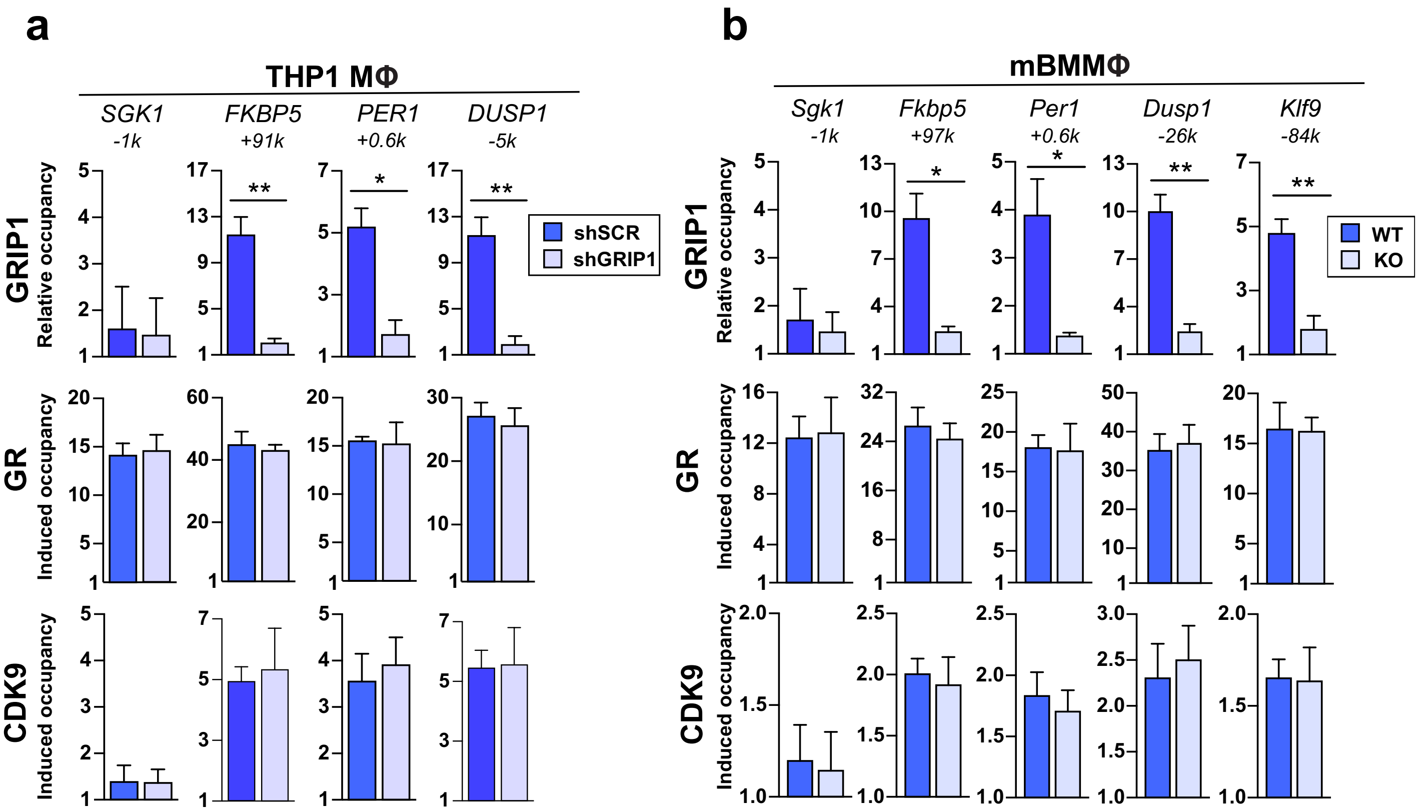

Supplementary Fig. 8 | GR and CDK9 recruitment is unaffected by GRIP1 levels.
GRIP1-sufficient (scrambled small hairpin, shSCR) and -depleted (shGRIP1) THP1 cells (**a**) and WT and GRIP1 KO BMMΦ (**b**) were treated with dexamethasone (Dex) for 1 h and GRIP1, GR and CDK9 occupancy at indicated GR binding sites was assessed by ChIP-qPCR as described in Fig. 1f. Shown are mean+SEM; n≥3 (* p<.05, ** p<.01; unpaired, two-tailed Student's t-test).

Supplementary Figure 9

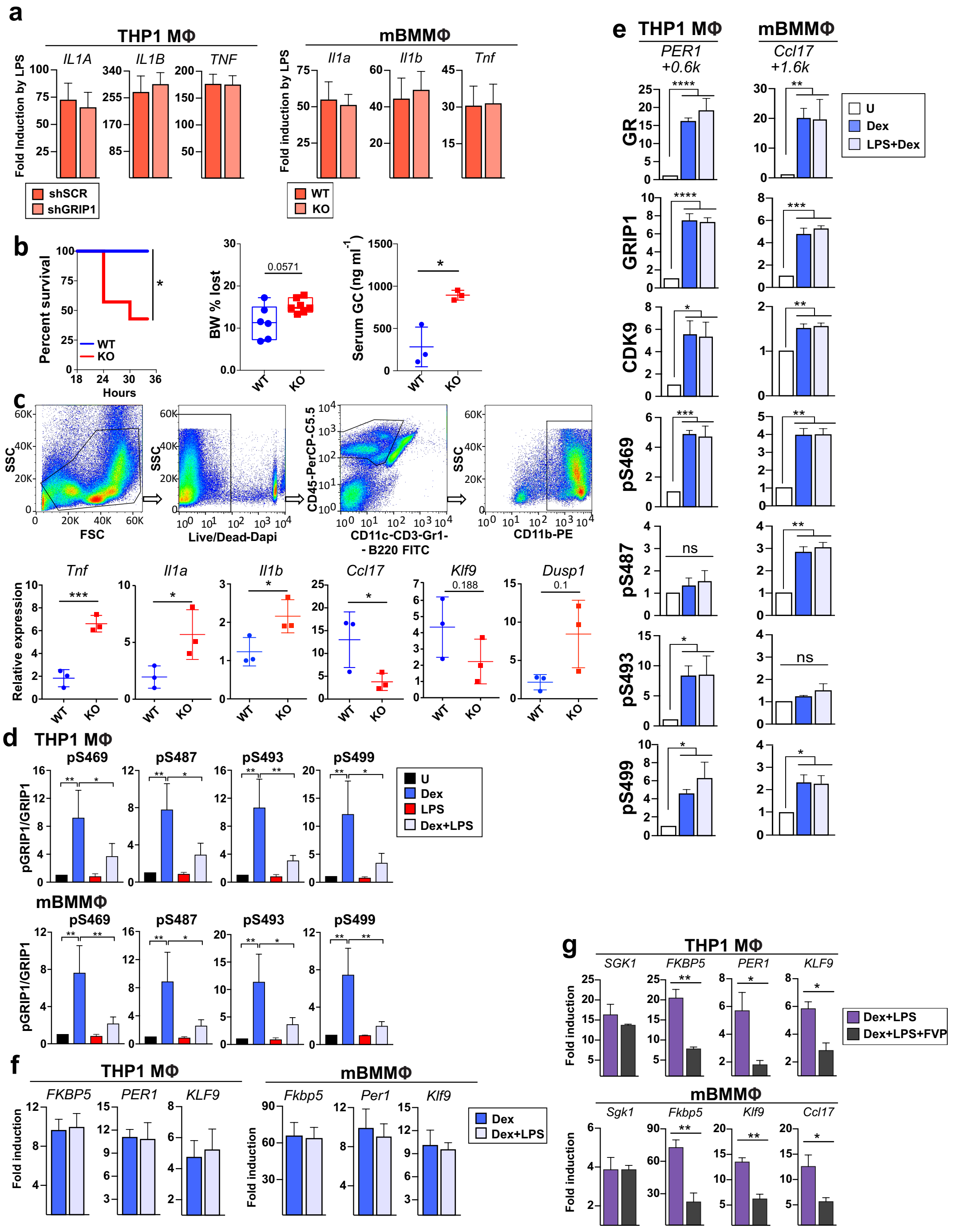

Supplementary Fig. 9 | The effect of lipopolysaccharide (LPS) on GRIP1 phosphorylation.
(**a**) THP1 MΦ (transduced with small hairpin scrambled shSCR or shGRIP1) or mouse WT or GRIP1 KO BMMΦ from Fig. 1a-b were treated with LPS (10 ng ml^{-1}, 1 h) and fold induction of indicated genes was measured in small by RT-qPCR as described in Fig. 1a (shown are mean+SD; n≥3; unpaired, two-tailed Student's t-test). (**b**) WT and GRIP1 KO mice were intraperitoneally injected with 5 mg kg^{-1} LPS and assessed for survival rate (left, n=6-7; Kaplan-Meier analysis and the log-rank test; * p<.05), percent of starting body weight (BW) loss at 24 h post-injection (middle, n=6-7; Mann-Whitney test), and serum GC levels at 12 h post-injection (right, n=3; * p<.05; unpaired, two-tailed Student's t-test). (**c**) Peritoneal macrophages were sorted from gavages by flow cytometry selecting Dapi-negative (live) cells that were negative for CD11c, CD3, Gr1, B220; but positive for CD45, CD11b (top, n=3). RNA was extracted from peritoneal MΦ and relative gene expression was compared between genotypes using β-actin expression for normalization and setting the lowest expression of a gene as control (=1, bottom; * p<.1, ** p<0.05, *** p<.01; unpaired, two-tailed Student's t-test). (**d**) Immunoblots from Fig. 6c were quantified as in Supplementary Fig. 5a. U, untreated; Dex, dexamethasone. Shown are mean+SEM; n≥3; One-Way ANOVA with Bonferroni's multiple comparison test. (**e**) Additional genes for Fig. 6d-e. THP1 MΦ and mouse BMMΦ treated with ±Dex ±LPS for 1 h were analyzed for GR, GRIP1, CDK9 and phospho-GRIP1 occupancy by ChIP-qPCR. Shown are mean+SD, n≥3; * p<.05, ** p<.01, *** p<.001, **** p<.0001, ns, non-significant; One-Way ANOVA with Dunnett's multiple comparison test. (**f**) THP1 MΦ and BMMΦ were treated for 2 h with Dex±LPS and fold induction of indicated was genes analyzed by RT-qPCR as described in Fig. 1a; shown are mean+SD; n≥3 (unpaired, two-tailed Student's t-test). (**g**) The GC-induction of indicated genes in the presence of LPS (2 h) was compared ± 50 nM flavopiridol (FVP) using β-actin as a housekeeping control for normalization and expressing the transcript level of each gene in the presence of Dex relative to that in untreated or FVP alone-treated cells (=1; n=3, * p<.05, ** p<.01; unpaired two-tailed Student's t-test).

Supplementary figure 10a

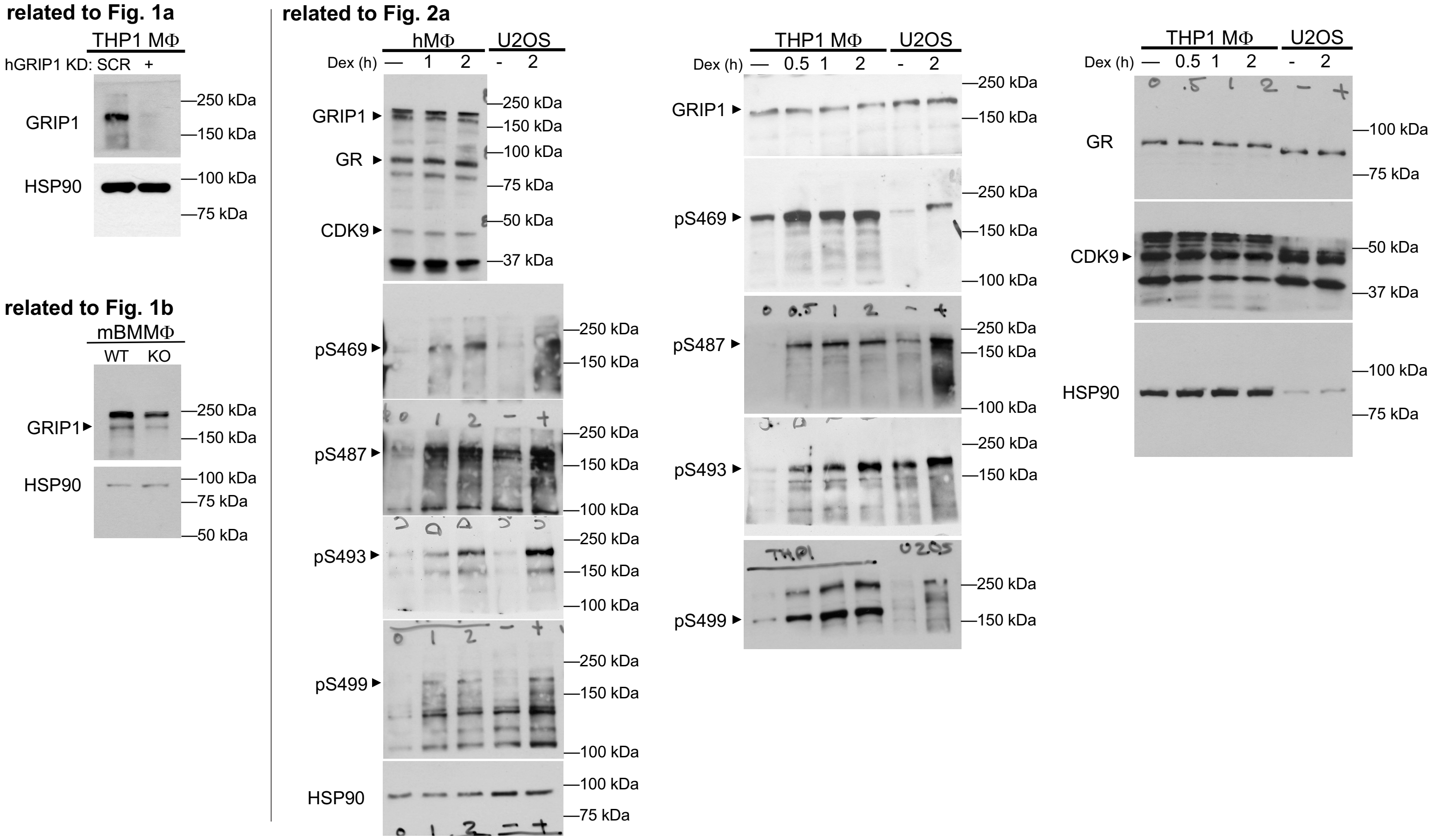

Supplementary Fig. 10 | Full-size western blot scans
(**a**) for western blots shown in figures 1a and 2a

Supplementary figure 10b

related to Fig. 2a

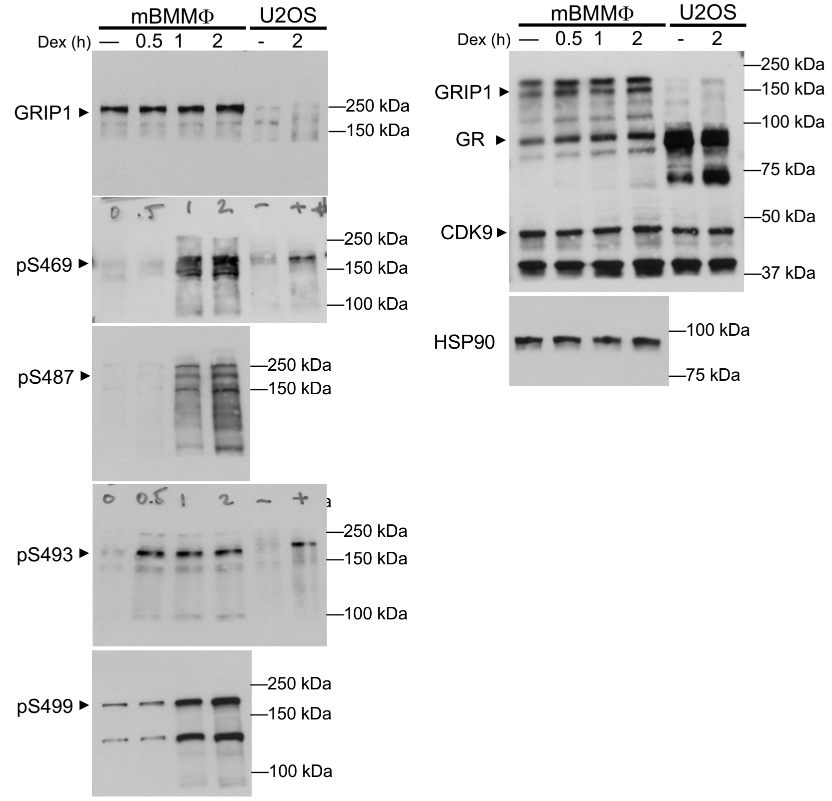

Supplementary Fig. 10 | Full-size western blot scans
(**b**) for western blots shown in figure 2a

Supplementary figure 10c

related to Fig. 2b

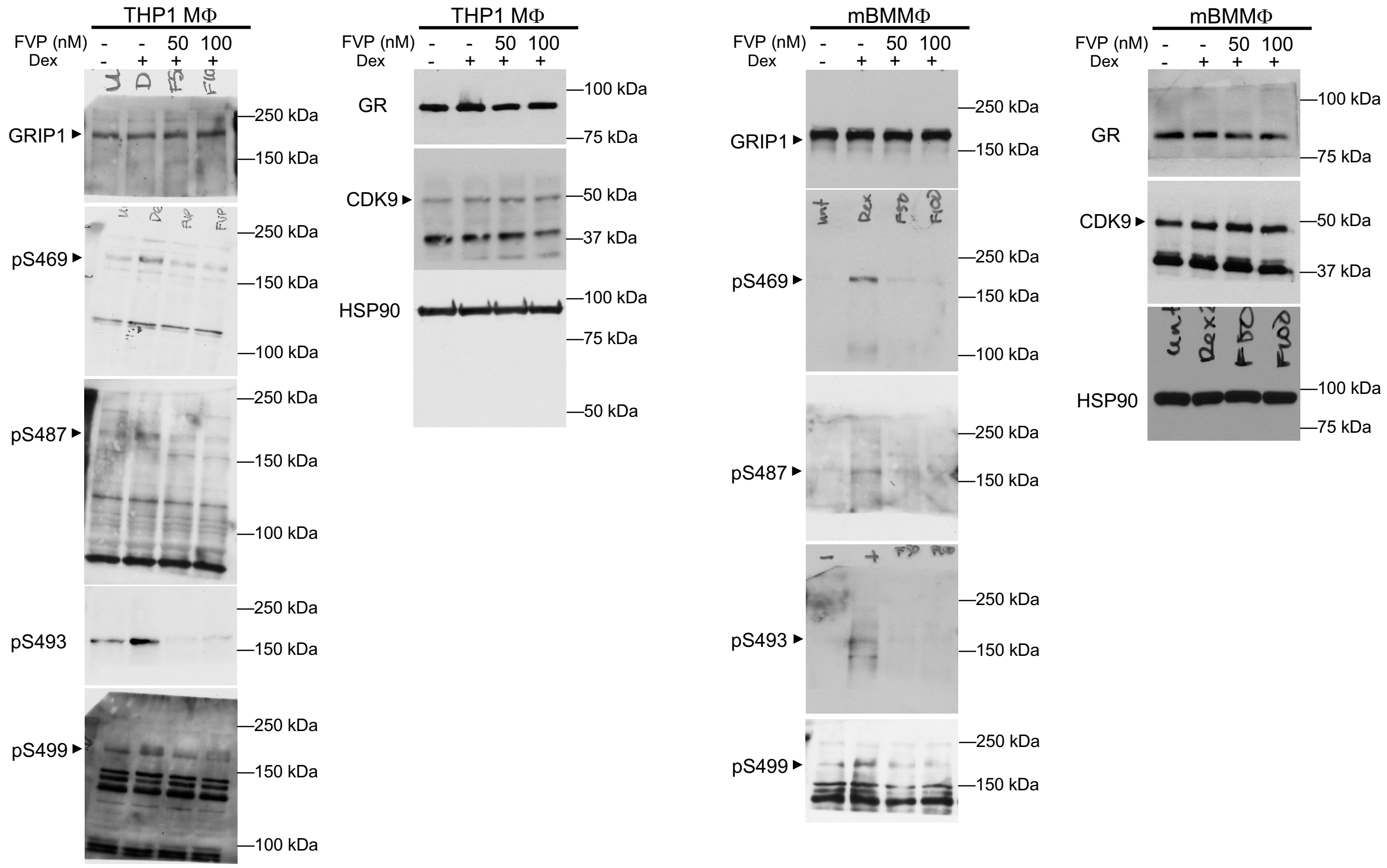

Supplementary Fig. 10 | Full-size western blot scans
(**c**) for western blots shown in figure 2b

Supplementary figure 10d

related to Fig. 2c

related to Fig. 2d

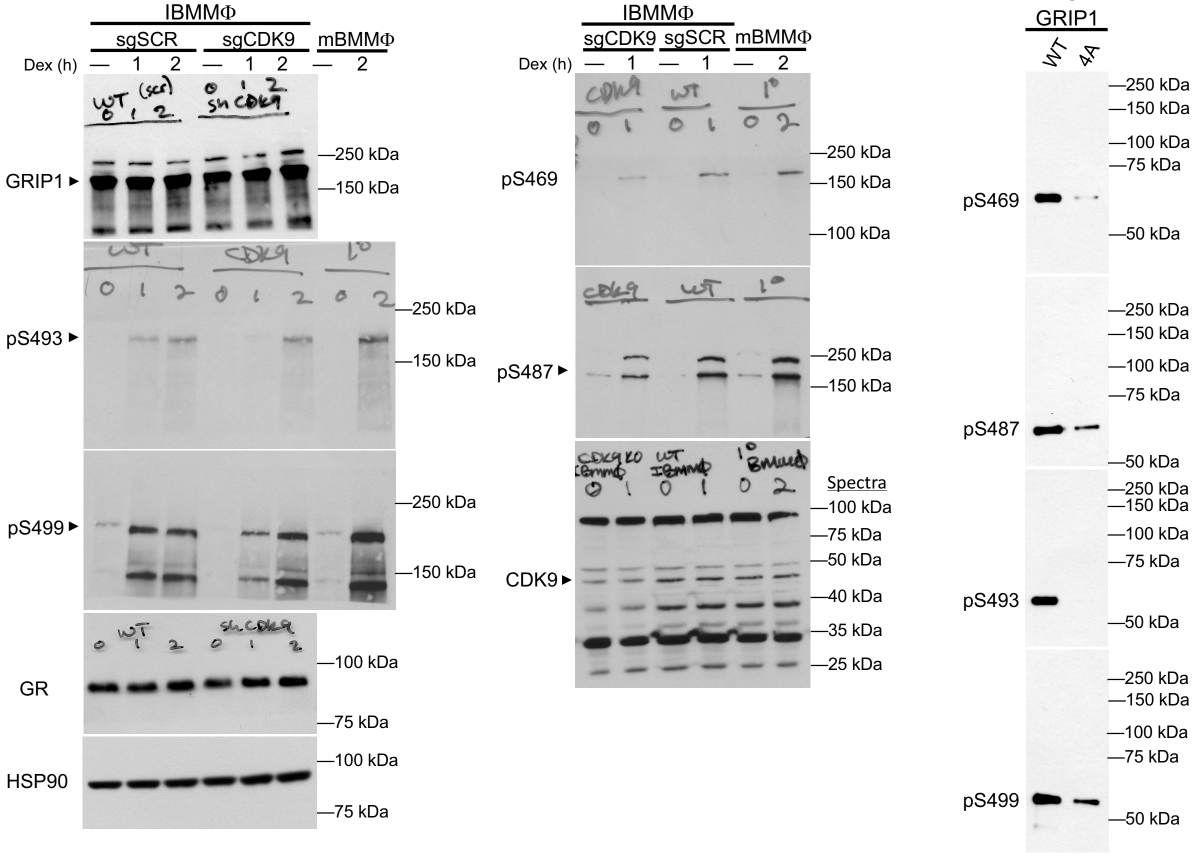

Supplementary Fig. 10 | Full-size western blot scans
(**d**) for western blots shown in figures 2c and 2d

related to Fig. 2e

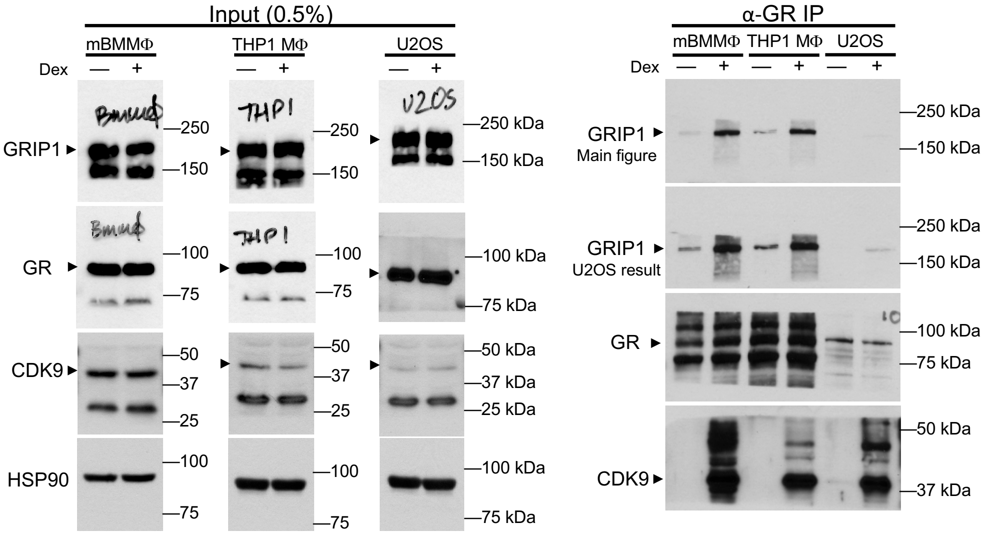

Supplementary Fig. 10 | Full-size western blot scans
(**e**) for western blots shown in figure 2e

Supplementary figure 10f

related to Supplementary Fig. 5d

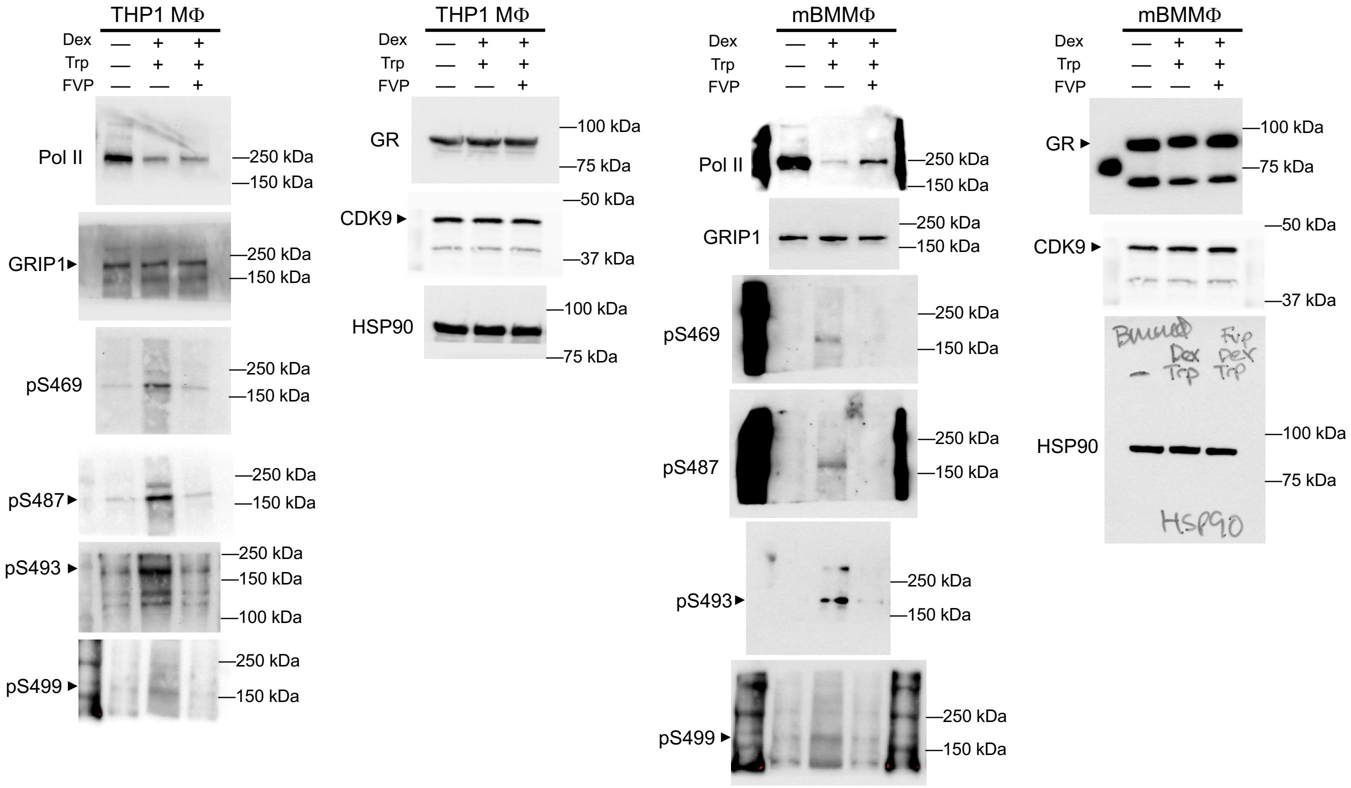

Supplementary Fig. 10 | Full-size western blot scans
(**f**) for western blots shown in Supplementary Fig 5d.

related to Fig. 3c

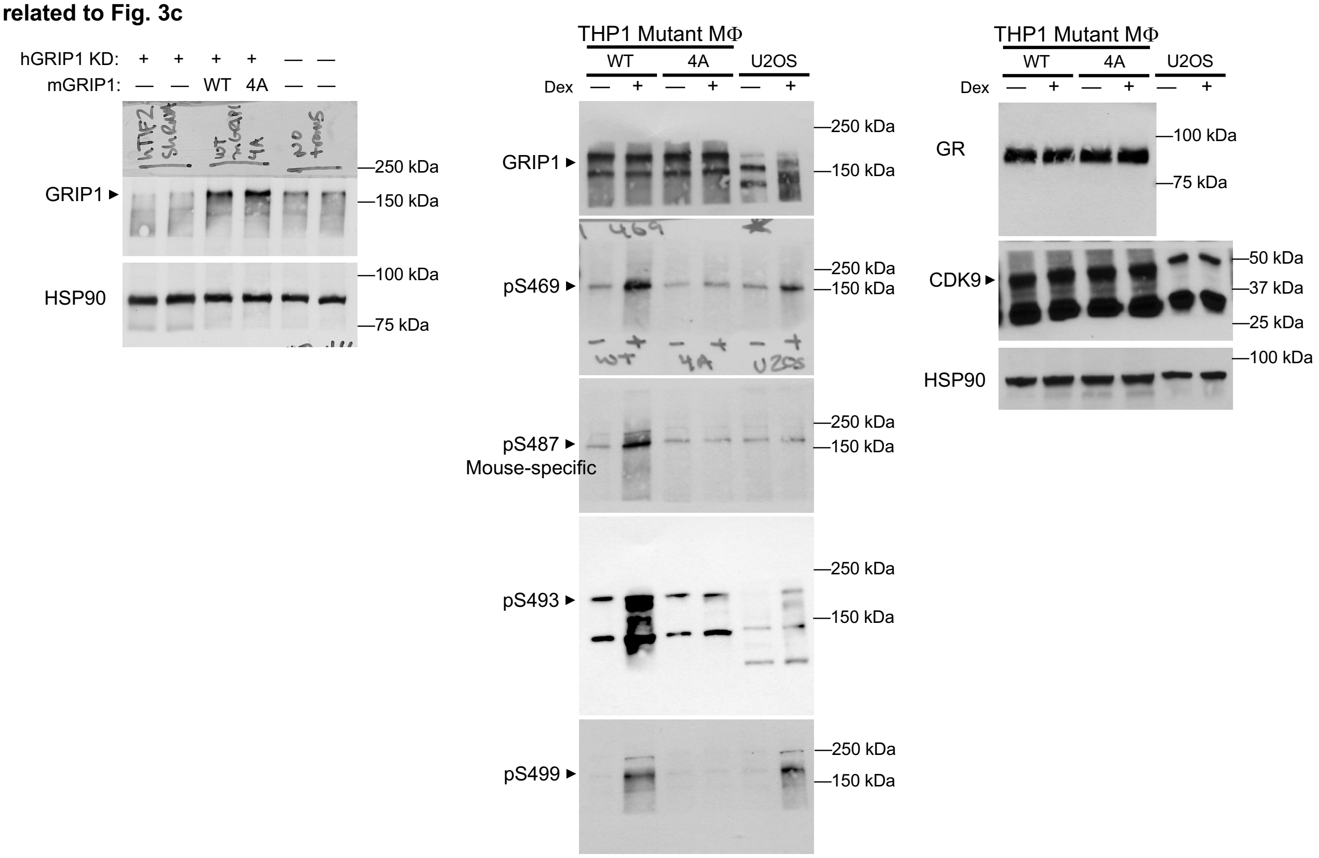

Supplementary Fig. 10 | Full-size western blot scans
(**g**) for western blots shown in figure 3c

Supplementary figure 10h

related Fig. 6c

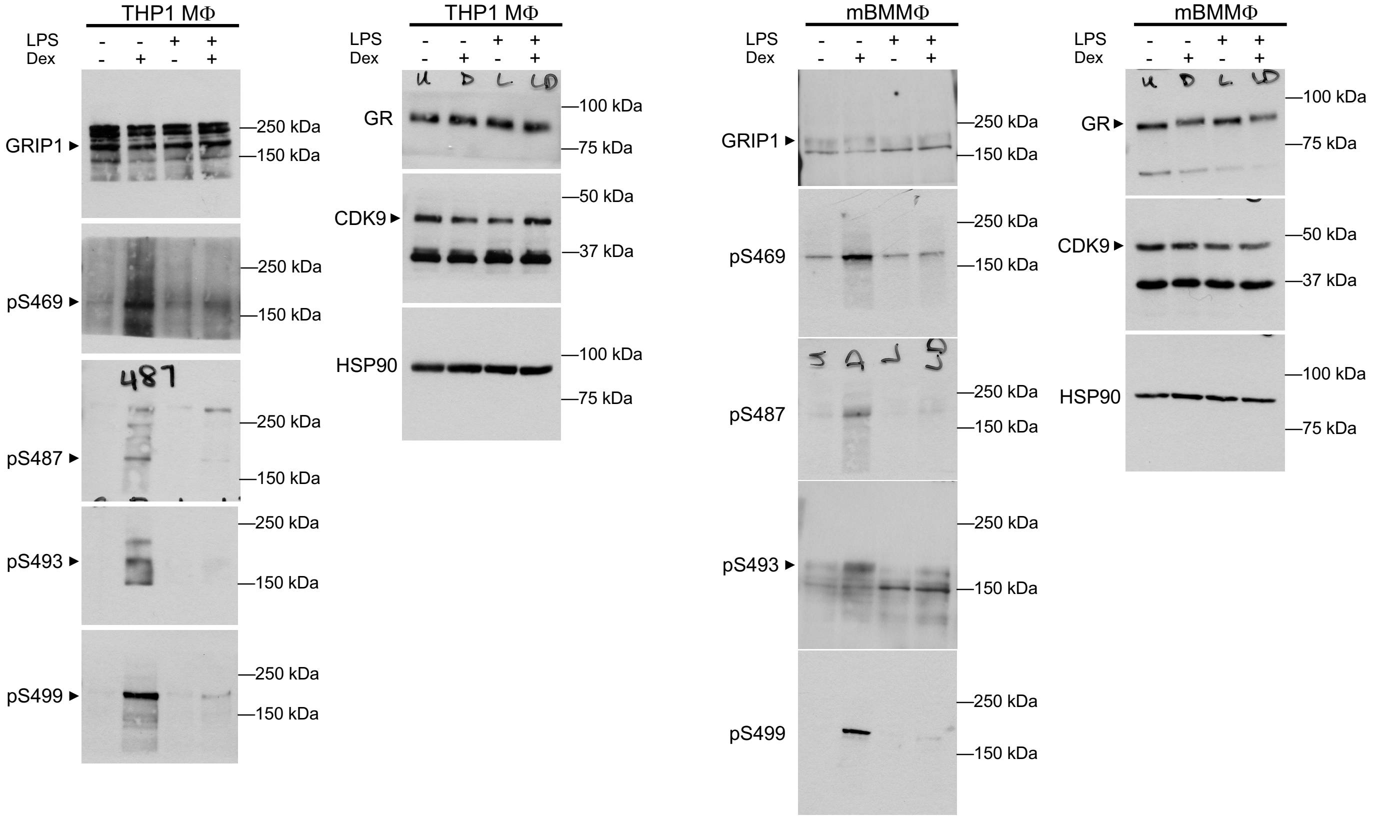

Supplementary Fig. 10 | Full-size western blot scans
(**h**) for western blots shown in figure 6

Supplementary Table 1. Primers used in this study.

**ChIP**	**Forward**	**Reverse**
28s	GATCCTTCGATGTCGGCTCTTCCTATC	AGGGTAAAACTAACCTGTCTCACG
**HUMAN**		
SGK -1k	TCCCTTCGCTTGTTACCTCCTCAC	CTGAGAACATTTTGTCCGTTCCGC
FKBP5 +91k	CTGCGCAATCGGAGTGTAAC	ATCGAGTTCATGTGCCAGCC
PER1 +0.6k	GCTGGTTCCTGCTGTTGGCCACA	CAGAAGACACACAACAGCCAACAGATC
DUSP1 -5k	GAGCTAGCTGCCATTGCACA	TTGGACACACAGCTCACACA
KLF9 +15k	TGGGTGTGTGTCTCTGACATC	GTCTGCTGGAACTGCATGTG
SGK1 TSS	CCTGCGCGACAGTGAGAAGT	TCTCAATGGGGACAGAACCG
FKBP5 TSS	CCTGTTTTCCCCAAATCTCA	CCCCAGAACAAACTCCTACC
PER1 TSS	GGAGCTTCCACTCGGCTGCG	GCAGAGATGCCTACCTGGTCGC
IL1A +1.5k	AGTTTGCTATGTACCTGTCTCCT	TGGGCACATAAGGAATACCAACA
IL1B -4.5k	GCCTGGCAGATTCACCTCTG	ATTCACACAGCACGTCACCG
**MOUSE**		
Sgk -1k	GAGAAACCCCTGCTCCCTCTAA	TCCGCATAAATTTTGAGCCTTGC
Fkbp5 +97k	GCACATCAAGTGAGTCTGGTCACTGC	TGCCAGCCACATTCAGAACAGGG
Per1 +0.6k	TACAGGACCGCTGTCGTTGGGTT	CGTGTTCTCTTGGCTGATGGCCC
Dusp1 -26k	CCACTAGATGAGCACTGATCAGCAG	GAGATTTGAGCTTCGAACAGAAGTTGGGT
Klf9 -84k	GCTCGTTGGACAAAGAGATGATG	CTGTGGTTGTTGTGGAACAGTTT
Ccl17 +1.6k	TAAAACGGCCTGTGACCAGC	AATGGAAGGCGTTCTGCACT
Sgk1 TSS	TCGCAACTCAGTCTACAGCC	AGACTGAGGGGAGCAGTGAA
Fkbp5 TSS	CCACCTCCCATAGGGCCA	CCACCAATCGGGACGGG
Per1 TSS	ATTATGCAACCCGCCTCCCA	TCCGGGACAAAGACTAACCC
Il1a -10k	GCGACCTCGAGTCAGTCCTCACT	AGCACCAGAAGTGACTCATCCTCCA
Il1b -3.1k	AGGATGGTGACGGGCACTCTAGC	CAGCTTTGAAGAAATGCCTGCCTCC
**RT-qPCR:**	**Forward**	**Reverse**
**HUMAN**		
ACTINB	TTGTTACAGGAAGTCCCTTGCC	ATGCTATCACCTCCCCTGTGTG
SGK1	CTATGCATGCAAACACCCTG	GCCAAGGTTGATTTGCTGAG
FKBP5	AGGGAGACTGCCAGCCGAGC	GGCATGGGACATTGGGGTGGC
PER1	ACTCCCCTATCCGCTTCTGTGCC	GGCCCAACACGAAGGCTACCTTG
DUSP1	GTGGTTGTCCTCCACAGGGATGC	GCCTTGGGCATCACTGCCTTGAT
KLF9	TTTTCCCGAGTCCACTGACG	CTGAGCAAGAGAATGCCGGA
A20(TNFAIP3)	GAAAGTCCCGTGGAAATCCC	GGGGTGTGATCTCTCTTGGC
IL10	GCTGGCCACAGCTTTCAAGA	TTCCAGTGTCTCGGAGGGAT
MT2A	GCAAATGCAAAGAGTGCAAA	ATCCAGGTTTGTGGAAGTCG
GILZ(TSC22D3)	AATGCGGCCACGGATGCCTTG	GGACTTCACGTTTCAGTGGACA
IL1A	GCTACTACCACCATGCTCTCC	AGGCTGCATGGATCAATCTGT
IL1B	CTGTGTCTCACTGGAAGAGGTTA	ACTATGGGTTTAACTCCCAACCC
TNF	CTGAACAATAGGCTGTTCCCATGTAGC	GGCTCAGCAATGAGTGACAGTTGG
**MOUSE**		
Actinb	AGGTGTGCACTTTTATTGGTCTCAA	TGTATGAAGGCTTTGGTCTCCCT
Sgk1	AAGACCTCACAAGCTCATTGAG	AGCTGACAGAACATTTTAAAAGA
Fkbp5	TGGGATCGACAAAGCCCTGGTGA	GCTCAGCATTGGGGTCAATGCCA
Per1	TGGACTCTGATATCCAGGAGCTCC	GGGGACATCAGAGGGCCAACTCCA
Dusp1	TTTGTGTAGGTCGGTGGTCTGCCTT	TGGCTTTGTCTGTCAGTGCCGAAAG
Klf9	CCCACTGTGTGAGAAGAGATTCAT	TCTTGATCATGCTGGGATGGAAC
Ccl17	CGGAACATTCCATGGGTCCTC	TTGAAGTAATCCAGGCAGCACTC
Il1a	GGAAGTGCTGACAGTCTGTATGTAC	GTGGCTCCACTAGGGTTTGCTC
Il1b	GGGCTGCTTCCAAACCTTTGACC	GTAGCTGCCACAGCTTCTCCACAGCC
Tnf	ACTCCAGCTGCTCCTCCACTTG	GCCTCCCTCTCATCAGTTCTATGG
